# High thermostability improves neutralizing antibody responses induced by native-like HIV-1 envelope trimers

**DOI:** 10.1038/s41541-022-00446-4

**Published:** 2022-02-28

**Authors:** Iván del Moral-Sánchez, Rebecca A. Russell, Edith E. Schermer, Christopher A. Cottrell, Joel D. Allen, Alba Torrents de la Peña, Celia C. LaBranche, Sanjeev Kumar, Max Crispin, Andrew B. Ward, David C. Montefiori, Quentin J. Sattentau, Kwinten Sliepen, Rogier W. Sanders

**Affiliations:** 1 Department of Medical Microbiology, Amsterdam UMC, University of Amsterdam, Amsterdam, Netherlands; 2 The Sir William Dunn School of Pathology, University of Oxford, Oxford, UK; 3 Department of Integrative, Structural and Computational Biology, The Scripps Research Institute, La Jolla, CA USA; 4 School of Biological Sciences, University of Southampton, Southampton, UK; 5 Department of Surgery, Duke University School of Medicine, Durham, NC USA; 6 Department of Biochemistry, All India Institute of Medical Sciences, New Delhi, India; 7 Department of Microbiology and Immunology, Weill Medical College of Cornell University, New York, NY USA

**Keywords:** Protein vaccines, Protein vaccines, HIV infections

## Abstract

Soluble HIV-1 envelope glycoprotein (Env) immunogens are a prime constituent of candidate vaccines designed to induce broadly neutralizing antibodies. Several lines of evidence suggest that enhancing Env immunogen thermostability can improve neutralizing antibody (NAb) responses. Here, we generated BG505 SOSIP.v9 trimers, which displayed virtually no reactivity with non-neutralizing antibodies and showed increased global and epitope thermostability, compared to previous BG505 SOSIP versions. Chemical crosslinking of BG505 SOSIP.v9 further increased the melting temperature to 91.3 °C, which is almost 25 °C higher than that of the prototype SOSIP.664 trimer. Next, we compared the immunogenicity of a palette of BG505-based SOSIP trimers with a gradient of thermostabilities in rabbits. We also included SOSIP.v9 proteins in which a strain-specific immunodominant epitope was masked by glycans to redirect the NAb response to other subdominant epitopes. We found that increased trimer thermostability correlated with increased potency and consistency of the autologous NAb response. Furthermore, glycan masking steered the NAb response to subdominant epitopes without decreasing the potency of the autologous NAb response. In summary, SOSIP.v9 trimers and their glycan masked versions represent an improved platform for HIV-1 Env based vaccination strategies.

## Introduction

One of the main goals of the HIV-1 vaccine field has been the design of soluble envelope glycoprotein (Env)-based immunogens that can induce protective neutralizing antibody (NAb) responses^1–4^. However, several intrinsic features of Env result in immune evasion by hindering the development of NAb responses^4^. For instance, the instability and conformational flexibility of the Env complex results in the exposure of immunodominant non-neutralizing antibody (non-NAb) epitopes that have been traditionally hypothesized to act as immune decoys^5^. These non-neutralizing epitopes include the immunodominant variable region 3 (V3), which is normally hidden inside the closed structure of native Env^6,7^, the inner domain of gp120^8,9^ and part of gp41. Furthermore, much of the protein surface that is exposed on native Env is either hypervariable or covered by a dense glycan shield^10–12^.

Several advances, exemplified by the BG505 SOSIP.664 prototype, allow for the routine generation of soluble Env trimers that closely mimic the prefusion conformation of viral Env^6,13–19^. The SOSIP.664 design contains an optimized furin cleavage motif (R6), an Ile-559-Pro (IP) substitution that stabilizes Env in its prefusion trimeric conformation and a disulfide bond (SOS) between positions 501 and 605 that covalently links the gp120 and gp41 subunits^13,20–22^. Incidentally, the “IP” substitution was, in a modified form, also applied to stabilized SARS-CoV-2 Spike protein in many COVID vaccines, termed “2P”^23,24^. Contrary to gp120 and non-native-like trimers, SOSIP.664 and other first-generation native-like trimers can induce NAb responses against autologous neutralization-resistant (Tier 2) viruses^6,25,26^. However, they still expose several non-NAb epitopes and induce significant V3-directed responses. Thus, a significant number of studies have focused on improving the performance of Env immunogens by immunosilencing undesired epitopes and reducing their conformational flexibility^4,27,28^. The stability derived from the reduced conformational flexibility might also result in a prolonged antigen presentation in the prefusion native-like conformation and thus increase the probability of inducing the desired NAb responses. Furthermore, more stable immunogens may facilitate a longer shelf-life and require less stringent cold-chain conditions, thus allowing their deployment under real-world conditions^29^.

Several stability-enhancing modifications to the BG505 SOSIP.664 prototype have been described. BG505 DS-SOSIP was generated by including an intra-gp120 disulfide bond (201C-433C) to prevent CD4-induced (CD4i) rearrangements that lead to non-NAb epitope exposure^30,31^. For the design of SOSIP.v4 trimers, we included two mutations (64K/66R and 316W), at positions neighboring the V3 loop, which resulted in reduced exposure of CD4i and V3 non-NAb epitopes and increased thermostability^32^. The MD39 set of mutations, discovered by directed molecular evolution, stabilize the core of Env trimers and result in improved trimerization and reduced V3 reactivity^33^. We further stabilized the prefusion sequestered conformation of the V3 loop by hydrophobic interactions induced by a 306L-308L pair of mutations^34^. Furthermore, we designed SOSIP.v6 trimers by introducing additional intra- (73C-561C) and interprotomer (49C-555C) disulfide bonds. SOSIP.v6 trimers showed significantly increased melting temperatures and induced strong autologous tier 2 NAb responses^35^. Chemical crosslinking with glutaraldehyde (GLA) and EDC/NHS has also been used to improve the half-life of Env immunogens and reduce their conformational flexibility. Crosslinked trimers induce more potent NAb responses than their non-crosslinked counterparts, indicating that higher stability is related to improved immunogenicity^36–38^.

The extensive glycosylation of the Env protein provides protection from NAbs targeting conserved regions^12,39,40^. Holes in this glycan shield expose strain-specific protein segments that lure the immune system into generating narrow-neutralizing antibody responses that might compete with responses targeting other, potentially cross-neutralizing, epitopes^41–44^. For instance, the strain-specific glycan hole around positions 241 and 289 in BG505 SOSIP trimers is immunodominant in rabbits and to a lesser extent in macaques^42,45–47^. The NAb responses directed to these glycan holes are of narrow specificity and unlikely to develop breadth^48^. They likely represent dead-end pathways on the route towards broadly neutralizing antibodies (bNAbs) and possibly compete with potential bNAb responses^49^. However, it has been shown that 241/289 glycan hole responses can be redirected by opening up other glycan holes in BG505 SOSIP trimers^50^.

Here, we combined several stabilizing mutations to generate a set of BG505 SOSIP trimers, named SOSIP.v9.1-v9.4, that display a high thermal stability and low reactivity to non-NAbs. Next, we immunized rabbits with a palette of BG505-based SOSIP trimers with a gradient of stabilities to determine whether greater immunogen stability improves the magnitude and consistency of the NAb response. Furthermore, we also immunized rabbits with ultrastable SOSIP.v9 trimers in which the 241/289 glycan hole was masked. We observed that this glycan masking efficiently redirected the NAb responses from the immunodominant 241/289 glycan hole to a more subdominant epitope (C3/465 region) without negatively affecting the magnitude of the NAb response. These ultrastable immunogens provide an improved design platform for epitope-focused vaccine strategies.

## Results

### Design and generation of hyperstable BG505 SOSIP trimers

**Fig. 1:**
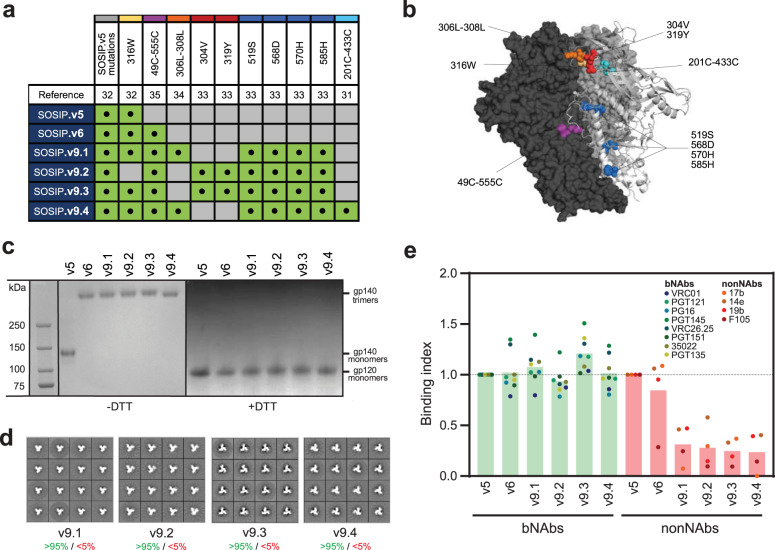
Design and biophysical characterization of ultrastable SOSIP.v9 trimers. **a** Table showing the combinations of stabilizing mutations used for the generation of SOSIP.v9 trimers. SOSIP.v5 mutations refer to 64K, 315Q, 501C-605C, R6, 535M, 543N, 559P and the truncation after residue 664^13,32^. **b** Location of the newly incorporated mutations in the three-dimensional structure of a BG505 SOSIP trimer. Mutations appear colored according to the color code in Fig. 1A. **c** SDS-PAGE analysis of PGT145-purified SOSIP.v5, SOSIP.v6 and SOSIP.v9 trimers under non-reducing (-DTT) and reducing (+DTT) conditions. **d** 2D class averages generated by negative-stain electron microscopy analysis of PGT145-purified SOSIP.v9 trimers. The percentage of native-like and non-native-like trimers are showed in green and red, respectively. **e** Ni-NTA-capture enzyme-linked immunosorbent assay with PGT145-purified SOSIP.v9 and the control (SOSIP.v5 and SOSIP.v6) proteins against a panel of bNAbs and non-NAbs. The binding index represents the average of duplicate measures and corresponds to the ratio between the 2G12 (loading control antibody)-normalized area under the curve (AUC) values of each protein and the 2G12-normalized AUC of the SOSIP.v5 reference protein. The bars represent the average of the binding indexes calculated for all the bNAbs (light green) and non-NAbs (light red).

We previously described the BG505 SOSIP.v6 immunogen^35^, a thermostable SOSIP trimer that contained additional inter- and intraprotomeric disulfide bonds and folded as a covalent trimer with a remarkably high thermostability. However, SOSIP.v6 still showed some reactivity to non-NAbs and was not produced as efficiently as preceding SOSIP versions. Our initial goal here was to generate hyperstable SOSIP trimers that do not present these drawbacks. Thus, we selected a set of mutations that have been shown to improve the antigenicity, stability, trimerization, and purification yields of HIV-1 Env trimers^31,33–35^ and introduced them in the BG505 SOSIP.v6 design in different combinations to generate BG505 SOSIP.v9.1-v9.4 (Fig. 1a, Fig. S1). A subset of the MD39 mutations (519S, 568D, 570H, and 585H)^33^ were introduced in all four SOSIP.v9.1 -v9.4 trimers in order to improve trimerization and purification yields. Newly incorporated mutations 306L-308L^34^, 304V and 319Y^33^, as well as the 316W mutation previously introduced in SOSIP.v4^32^, sequester the immunodominant V3 loop region by hydrophobic interactions. Due to the proximity of these mutations in the three-dimensional structure of the trimer (Fig. 1b), we included them in three different combinations (316W+306L-308L in SOSIP.v9.1 and SOSIP.9.4, 304V+319Y in SOSIP.v9.2 and 316W+304V+319Y in SOSIP.v9.3) to avoid undesired interactions or interferences. The disulfide bond 201C-433C, which reduces sampling of the CD4-induced state^31^, was introduced in SOSIP.v9.4 only.

We first purified His-tagged versions of BG505 SOSIP.v9.1-v9.4 expressed in HEK293F suspension cells by PGT145 affinity chromatography, as previously described^32,51^. All SOSIP.v9.1-v9.4 proteins showed increased purification yields compared to their SOSIP.v6 predecessor, with a 3.8 - 4.3-fold increase in the case of SOSIP.v9.1-v9.3 and a more subtle but consistent ~1.3-fold increase for SOSIP.v9.4 (Table 1). These yields were ~1.5-fold higher than those of non-hyperstabilized SOSIP.v4 and SOSIP.v5 trimers^31^. As expected, after PGT145 purification, all SOSIP versions tested formed trimers, as assessed by the BN-PAGE analysis (Fig. S2). An SDS-PAGE analysis showed that all SOSIP.v9.1-v9.4 purified proteins remain trimeric under non-reducing conditions, while they separate into monomers under reducing conditions (Fig. 1c). This confirms that SOSIP.v9 trimers contain interprotomer disulfide bonds that covalently link the three protomers, similarly to the SOSIP.v6 predecessor^35^. Furthermore, 2D class averages of negative-stain electron microscopy (NS-EM) images revealed that all four SOSIP.v9 variants assumed a >95% native-like conformation (Fig. 1d, Table 1). A Ni-NTA ELISA experiment with the purified proteins revealed a favorable antigenic profile for all the SOSIP.v9 trimers compared to SOSIP.v5 and SOSIP.v6 (Fig. 1e, Fig. S3), with similar (or higher) binding to bNAbs and decreased binding to non-NAbs. BG505 SOSIP.v9.3 showed the most desirable antigenic profile, with increased binding to all the bNAbs tested, in particular the quaternary-dependent bNAbs PG16, PGT145, and VRC026.25, and decreased binding to all non-NAbs tested (17b + sCD4, 14e, 19b, and F105) (Fig. 1e, Fig. S3). In summary, the four BG505 SOSIP.v9 proteins fold as covalent native-like Env trimers and are improved compared to SOSIP.v6 in terms of production yields and antigenicity. Overall, the SOSIP.v9.3 construct displayed the most promising features, especially regarding its antigenicity profile.

**Table 1:** Production yields and biophysical characterization of SOSIP.v9 trimers.

SOSIP version	Yield^a^ (mg/L) (≈)	Native-like (%) (>)	*T*_m_ (°C)			Δ*T*_m_ (°C)
			2G12	PGT145	DSF	
SOSIP**.664**	2.0^b^	95^b^	NA	NA	67.6^b^	0.0
SOSIP**.v4**	2.0^b^	95^b^	NA	NA	70.7^b^	+3.1
SOSIP**.v5**	2.0^b^	95^b^	69.7	66.9	74.2	+6.6
SOSIP**.v6**	0.8^b^	95^b^	73.3	71.4	77.3	+9.7
SOSIP**.v9.1**	3.0	95	75.9	74.6	80.2	+12.6
SOSIP**.v9.2**	3.1	95	76.0	74.0	79.7	+12.1
SOSIP**.v9.3**	3.4	95	78.3	76.1	80.7	+13.1
SOSIP**.v9.4**	1.0	95	85.0	82.0	84.0	+16.4
SOSIP**.v9.3.GM**	2.4	95	NA	NA	79.9	+12.3
SOSIP**.v9.4.GM**	0.6	85	NA	NA	83.2	+15.6
SOSIP**.v9.3.XL**	NA	95	NA	NA	91.3	+23.7

^a^Values correspond to the mean of the yields of at least two independent purifications.

^b^Data from Torrents de la Peña et al. Cell Reports 2017^35^.

All the data were derived from PGT145-purified His-tagged proteins, except for the ones corresponding to the untagged SOSIP.v9.3.XL trimer, which was purified as described in the methods sections. An overview of the modifications included in the design of SOSIP.v9 trimers and an alignment with previous SOSIP versions are shown in Fig. 1a and Fig. S1, respectively.

### SOSIP.v9 trimers show superior global and epitope-specific thermostability

**Fig. 2:**
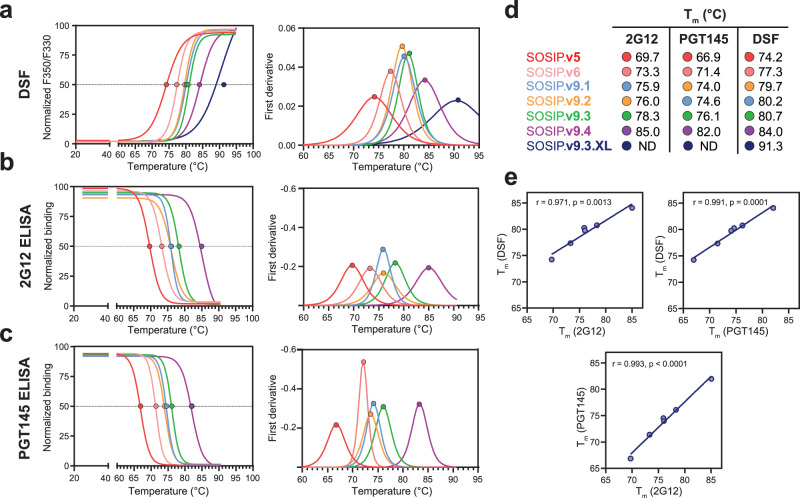
Thermostability of SOSIP.v9 and crosslinked SOSIP.v9.3 trimers. **a–c** Data used to determine the temperature of melting (*T*_m_) values of PGT145-purifed SOSIP.v9 trimers by nanoDSF (**a**), 2G12 thermostability ELISA (**b**) and PGT145 thermostability ELISA (**c**) assays. Left graphs show the normalized fluorescence ratio (F350/F330), 2G12 binding and PGT145 binding signals measured at different temperatures. Graphs on the right show the first derivative of the measured signals. Dots represent the *T*_m_ value of each protein, calculated as the temperature at which the signal is half of the maximum signal (left) or the value of the first derivative is maximized (right). **d** Temperatures of melting calculated for each protein and thermostability assay, ordered from lower to higher values. **e** Correlation between *T*_m_ values determined using the different thermostability assays. Pearson r and p-values are indicated for each pair of assays.

In order to evaluate the influence of the stabilizing mutations incorporated on the thermostability of SOSIP.v9 trimers, we used Nano Differential Scanning Fluorimetry (nanoDSF), which provides information on the overall thermostability, but does not reveal how stable individual bNAb epitopes are. The SOSIP.v9.1-v9.4 trimers presented superior global thermostability compared to all their precursors, including the hyperstable SOSIP.v6 (Fig. 2a, Table 1). The SOSIP.v9.3 and SOSIP.v9.4 proteins displayed the highest melting temperatures (*T*_m_) among the SOSIP.v9 variants, with *T*_m_ values of 80.7 °C and 84 °C, respectively (Fig. 2d, Table 1). These *T*_m_ values are 13.1 °C and 16.4 °C higher than the ones of first-generation BG505 SOSIP.664 trimers^35^.

To gauge the thermostability of specific bNAb epitopes, we performed thermostability ELISA assays with bNAb 2G12 (Fig. 2b), which depends on conformational structure but not on quaternary structure, and PGT145 (Fig. 2c), which depends on proper quaternary conformation at the trimer apex. All SOSIP.v9 trimers displayed more stable 2G12 and PGT145 epitopes than the previous SOSIP versions (Fig. 2b–d, Table 1). The T_m_ values of the 2G12 and PGT145 epitopes of SOSIP.v9.3 (78.3 °C and 76.1 °C, respectively) and SOSIP.v9.4 (85.0 °C and 82.0 °C, respectively) were the highest among the SOSIP.v9 proteins.

The results of all three techniques (nanoDSF, 2G12, and PGT145 thermostability ELISA) were strongly correlated (Fig. 2e, Table 1). The T_m_ values measured by nanoDSF were ~2 °C higher than the ones measured by 2G12 thermostability ELISA, and ~5 °C higher than those measured by PGT145 thermostability ELISA. These trends are expected, as trimer dissociation probably precedes loss of gp120 conformation and, finally, unfolding.

### Chemical crosslinking further stabilizes SOSIP.v9.3

To add an additional layer of stabilization, we used glutaraldehyde (GLA) to chemically crosslink the SOSIP.v9.3 immunogen (SOSIP.v9.3.XL)^37^. After purification of the SOSIP.v9.3.XL protein by PGT151 affinity chromatography, we visualized it by NS-EM, confirming that >95% of the species presented a native-like conformation (Fig. S4). Trimer antigenicity was evaluated in a BLI experiment with a small panel of relevant Abs (Fig. S5). Chemically crosslinked and PGT151-purified SOSIP.v9.3 showed similar 2G12 and PGT151 binding compared to normal SOSIP.v9.3, whereas PGT145 and VRC01 binding was decreased, consistent with previous crosslinking experiments^37,52^. The decrease in PGT145 binding has previously been hypothesized to be related to the direct modification of the lysine residues 168, 169, and 171, rather than to a loss of native-like apex conformation^37^. Notably, chemical crosslinking increased the T_m_ of SOSIP.v9.3 from 80.7 °C to 91.3 °C, which is an increase of 23.7 °C compared to the SOSIP.664 prototype^35^ (Fig. 2d, Table 1). Thus, we generated a panel of SOSIP trimers with a gradient of stabilities (SOSIP.v9.3.XL > SOSIP.v9.4 > SOSIP.v9.3 > SOSIP.v9.2 ≃ SOSIP.v9.1 > SOSIP.v6 > SOSIP.v5 > SOSIP.v4 > SOSIP.664) (Fig. 2d, Table 1).

### Addition of PNGS at position 241 and 289 fills the 241/289 glycan hole on SOSIP.v9 trimers

**Fig. 3:**
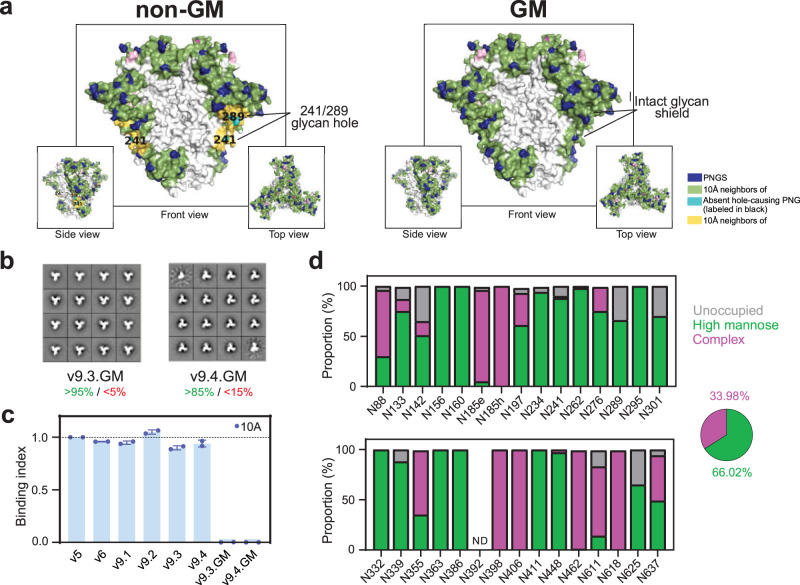
Design and characterization of glycan masked SOSIP.v9 trimers. **a** Representation of the glycan shield of non-glycan masked (non-GM), containing the 241/289 glycan hole (left), and glycan-masked (GM) SOSIP.v9 trimers, which present an intact glycan shield in the 241/289 region (right). Modeling of the glycan shields was performed using the Glycan Shield Mapping tool^41^. **b** 2D class averages obtained by negative-stain electron microscopy analysis of PGT145-purified SOSIP.v9 glycan masked trimers. The percentages of native-like and non-native-like trimers are indicated in green and red, respectively. **c** Reactivity of the 241/289 targeting mAb 10A^42^ against non-GM and GM SOSIP.v9 proteins measured by a Ni-NTA-capture ELISA. The binding index for each tested protein represents the values of duplicate measures and corresponds to the ratio between the 2G12 (loading control antibody)-normalized area under the curve (AUC) values of each protein and the 2G12-normalized AUC of the SOSIP.v5 reference protein. The bars and error bars represent the average and standard deviation of the ratios obtained for each of the experiments. **d** Site-specific glycan analysis of PGT145-purified SOSIP.v9.3.GM protein.

Considering the immunodominance of the N241/N289 BG505 glycan hole, we aimed to immunosilence this epitope and redirect the NAb responses towards other, potentially non-strain-specific, epitopes. Thus, we created Glycan hole Masked (GM) versions of the SOSIP.v9.3 and SOSIP.v9.4 trimers (SOSIP.v9.3.GM and SOSIP.v9.4.GM) by introducing PNGS at positions 241 and 289 (241N, 291S) together with changes that counteract the destabilizing effects result of the incorporation of such PNGS motifs (240T, 271I, 288L, 290E)^45^ (Fig. 3a).

We expressed the His-Tagged versions of SOSIP.v9.3.GM and SOSIP.v9.4.GM in 293 F suspension cells and purified them by PGT145 affinity chromatography. SOSIP.v9.3.GM and SOSIP.v9.4.GM showed slightly decreased purification yields (2.4 mg/L and 0.6 mg/L, respectively) as compared to the respective parental proteins (Table 1). They also expressed as covalently linked trimers, as observed on SDS-PAGE (Fig. S6). When imaged by NS-EM, we observed a reduction in the percentage of native-like trimers of SOSIP.v9.4.GM ( > 85% vs >95%), while SOSIP.v9.3.GM conserved a >95% native-like conformation (Fig. 3b, Table 1). Site-specific glycan analysis revealed that PNGS occupancy and glycan composition was similar for SOSIP.v9.3.GM (Fig. 3d, Fig. S7) when compared to previous SOSIP versions. The occupancy at the newly incorporated 241 and 289 PNGS was 90% and 66%, respectively, thus effectively filling the hole with at least one glycan in the majority of molecules (Fig. S7).

Both GM trimers showed an overall similar antigenic profile as SOSIP.v9.3 and SOSIP.v9.4 (Fig. S8). Furthermore, an antibody targeting the 241/289 glycan hole of BG505 (10 A)^42^ showed negligible reactivity to GM trimers, confirming the effective filling of the 241/289 hole (Fig. 3d).

The inclusion of new PNGS slightly influenced thermostability, with nanoDSF-measured T_m_ values of 79.9 °C and 83.2 °C for SOSIP.v9.3.GM and SOSIP.v9.4.GM, respectively (i.e. 0.8 °C lower compared to the parental proteins) (Fig. S9).

### Ultrastable SOSIP.v9 trimers induce autologous NAb responses

**Fig. 4:**
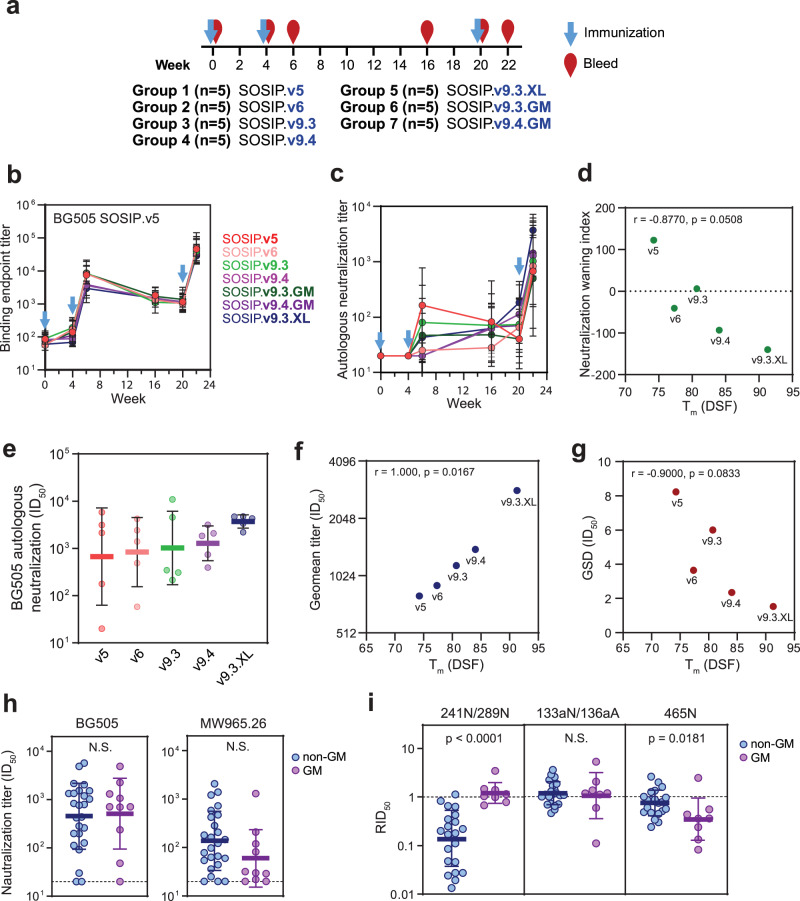
Immunogenicity of SOSIP.v9 proteins in rabbits. **a** Rabbits were immunized with different BG505 SOSIP trimer versions adjuvanted with squalene emulsion at weeks 0, 4 and 20. Antibody responses were evaluated at weeks 0, 4, 6, 16, 20, and 22. **b** Endpoint antibody binding titers over time against BG505 SOSIP.v5 trimer as measured by Ni-NTA ELISA. The mean binding titers and standard deviation of each group are shown. No significant differences were found by a Kruskal–Wallis statistical test between groups at any timepoint tested. **c** Midpoint neutralization titers (ID_50_) over time for sera of the immunized rabbits against a virus pseudotyped with a cytoplasmatic tail truncated BG505 Env (BG505.dCT). ID_50_ values were determined by TZM-bl assay and are detailed in Fig. S13. The geometric mean binding titers and geometric standard deviation of each group are represented **d** Correlation between the melting temperature of immunogens and the waning of autologous neutralization responses between weeks 6 and 20. The waning index corresponds to the difference between the ID_50_ values at weeks 6 and 20. The dotted line separates groups that show a reduction (positive waning values) or increase (negative waning values) in titers between these timepoints. Pearson test r and p correlation values are presented. **e** Midpoint neutralization titers (ID_50_) for week 22 sera of the immunized rabbits against an autologous BG505.dCT pseudovirus. Geometric means and geometric standard deviations are represented. **f**–**g** Effect of the ultrastabilization on the potency and consistency of autologous NAb responses. Correlation plots between geometric mean (Geomean) (**f**) and geometric standard deviation (GSD) (**g**) of the ID_50_ against the BG505 autologous pseudovirus and the melting temperatures (*T*_m_) of the immunogens, determined by nanoDSF. Spearman r and p-values are presented. **h**–**i** Effect of the 241/289 glycan hole masking on the autologous NAb responses. **h** Comparison of the neutralization titers elicited by non-glycan masked (non-GM) and glycan masked (GM) SOSIP.v9.3 and SOSIP.v9.4 immunogens against autologous BG505/T332N and Tier 1A MW965.26 pseudoviruses. ID_50_ values were determined in a TZM-bl assay and are presented in Fig. S15. No significant differences (N.S.) were found by a Mann–Whitney statistical test. **i** Reduction of the NAb titers against a panel of mutant (241/289N, 133aN/136aA, and 465N) BG505 pseudoviruses compared to the wild-type BG505/T332N pseudovirus. Relative ID_50_ (RID_50_) is calculated as the ratio between the ID_50_ against each mutant virus and the ID_50_ of the wild-type virus. Horizontal lines and error bars represent the geometric mean and geometric standard deviations. A dotted line is used to indicate the RID_50_ value for which there is no reduction or increase of ID_50_ values for the mutants.

To evaluate the immunogenicity of the SOSIP.v9 trimers and gauge their ability to generate NAb responses, we performed an immunization experiment in rabbits (Fig. 4a). We selected the following immunogens to be tested: (1) the BG505 SOSIP.v5 and SOSIP.v6 predecessors, to serve as control groups; (2) BG505 SOSIP.v9.3 and SOSIP.v9.4, as they showed the best antigenic profile and thermostability, respectively, among the SOSIP.v9 trimers; (3) BG505 SOSIP.v9.3.XL trimer, i.e. the trimer with the highest thermostability; and 4) glycan masked versions of BG505 SOSIP.v9.3 and SOSIP.v9.4 (SOSIP.v9.3.GM and SOSIP.v9.4.GM), to study redirection of responses away from the 241/289 hole. Untagged versions of these immunogens were purified by PGT145 affinity chromatography and an additional size exclusion chromatography (SEC) purification step to remove aggregated species. BN- and SDS-PAGE analyses were performed for quality control (Fig. S10).

Seven groups of animals (5 animals per group) were immunized at three timepoints (weeks 0, 4 and 20) with 30 µg of each of the test proteins formulated with Squalene Emulsion (SE) adjuvant. Sera were obtained from the rabbits at weeks 0, 4, 6, 16, 20 and 22 to characterize the antibody responses (Fig. 4a).

First, we measured the binding of the sera at the different timepoints to a His-tagged BG505 SOSIP.v5 trimer using a Ni-NTA ELISA assay. As expected, substantial increases in binding titers occurred at week 6 and week 22, two weeks after the second and third immunizations, respectively (Fig. 4b). We observed no significant differences in the binding titers induced by the different immunogens (Fig. S11a) at any timepoint. There were also no differences between the binding titers induced by the GM immunogens and their parental constructs (Fig. S11b).

Next, we measured the development of the NAb response against an autologous BG505 pseudovirus (Fig. 4c, Fig. S12). We observed that these autologous NAb responses evolved differently between weeks 6 and 20 for the different groups (Fig. 4c, Fig. S13). In some groups, in particular, the ones receiving SOSIP.v5, SOSIP.v9.3 and SOSIP.v9.3.GM, NAb responses peaked at week 6 and then declined resulting in lower NAb responses at week 20. In contrast, the NAb titers increased from week 6 to week 20 in the groups receiving SOSIP.v6, SOSIP.v9.4, SOSIP.v9.4.GM and SOSIP.v9.3.XL (Fig. 4c, Fig. S13). As a result, there was a trend that the groups receiving more stable proteins maintained higher NAb titers until week 20 (Fig. 4d). While this trend was not statistically significant, it would be consistent with the hypothesis that more stable immunogens might persist for longer thereby enhancing the durability of the response. Animals vaccinated with SOSIP.v6, SOSIP.v9.4 and SOSIP.v9.4.GM showed delayed responses, with no NAb activity detected at week 6 (two weeks after the second immunization), while a NAb response was detected by that time in the other rabbit groups. Nonetheless, all immunogens showed similar responses at week 16 and developed strong autologous NAb responses at week 22, after the third immunization (Fig. 4c, Fig. S12).

To assess the breadth of the NAb responses, we evaluated the neutralization of a panel of Tier 1 and Tier 2 heterologous viruses, including the viruses from the global HIV-1 panel^53^, at week 22 (Fig. S14). Except for some low titer responses, no consistent Tier 2 heterologous neutralization was observed. Remarkably, rabbits vaccinated with SOSIP.v9.4 and SOSIP.v9.4.GM immunogens showed significantly decreased NAb titers to the Tier 1 A MW965.26 virus (Fig. S15). Neutralizing responses to this virus are dominated by V3-targeting NAbs that are unable to neutralize Tier 2 primary HIV-1 isolates^54^, and the V3 might be less accessible in the SOSIP.v9.4 and SOSIP.v9.4.GM trimers as a consequence of the stabilization. Hence, NAb responses induced by these immunogens might be less hindered by competing V3-targeting narrow-NAb responses.

### Autologous NAb responses correlate with SOSIP trimer stability

To assess the influence of the thermostability of the immunogen on the induction of autologous NAb responses, we used the neutralization titers determined for the BG505 pseudovirus virus at week 22 (Fig. 4e, Fig. S12). When we plotted the autologous NAb titers, expressed as the geometric mean (Geomean) of the ID_50_ values of each group, versus the nanoDSF-measured *T*_m_ values of the corresponding immunogen, we observed a positive correlation (*p* = 0.0167), indicating that increased thermostability is associated with the induction of stronger NAb responses (Fig. 4f). We also observed an inverse trend (*p* = 0.0833) between the nanoDSF-measured T_m_ values and the spread of the ID_50_ values within each group of animals, which suggests that higher thermostability increases the consistency of the NAb response (Fig. 4g). These trends were also observed when 2G12 and PGT145 thermostability ELISA-measured *T*_m_ values were used (Fig. S16a) and were found irrespective of the presence or absence of the cytoplasmic tail in the BG505 gp160 used for pseudovirus production (Fig. S16b).

### Glycan masking redirects immunodominant glycan-hole responses to subdominant neutralizing epitopes

SOSIP.v9.3.GM and SOSIP.v9.4.GM immunogens were included in the rabbit immunization experiment to test the potential of the glycan masking strategy to redirect the NAb responses towards epitopes different than the 241/289 glycan hole. Surprisingly, we found that animals vaccinated with the glycan masked versions of SOSIP.v9.3 and SOSIP.v9.4 displayed autologous NAb responses of similar magnitude compared to those vaccinated with conventional SOSIP.v9.3 and SOSIP.v9.4, despite the lack of the dominant 241/289 NAb epitope (Fig. 4h). To map the neutralizing epitope of these animals we compared the ID_50_ titers of wild-type BG505 pseudovirus to the ID_50_ titer of a panel of mutant BG505 variants that contain PNGS sites at positions 241 and 289 (“241N/289N”), in the variable loop 1 at positions 133 and 136 (“133aN/136aA”) and at position 465 in the constant region 3 (465N) (Fig. S17). The relative ID_50_ titers (RID_50_) revealed that responses of most BG505 SOSIP-vaccinated rabbits induced a response to the immunodominant 241/289 glycan hole, consistent with previous studies^42,55^. However, few, if any, of NAbs elicited in recipients of GM-modified SOSIP trimers focused on the 241/289 glycan hole (*p* < 0.0001). Instead, NAb responses from these animals were significantly more focused on the 465 position than those in the non-GM groups (*p* = 0.0181) (Fig. 4i, Fig. S17). There was no difference for other epitopes, such as the one involving positions 133 and 136 (Fig. 4i). This indicates that the glycan masking strategy efficiently redirected the responses from the immunodominant 241/289 epitope to the subdominant C3/465 epitope, and possibly to other epitopes.

While the autologous neutralization in most animals could be attributed to Abs targeting either the 241/289 hole or the C3/465 epitope, we used a wider panel of mutant viruses to map the responses in animals in which these epitopes were not the major targets (i.e. animals UA139, UA142, UA155, and UA157). Only rabbit UA142 showed decreased neutralization to one of the mutants in the panel (BG505 G458Y), pointing at a CD4bs-targeting NAb response (Fig. S18). The NAb targets in the other three animals remain unidentified.

## Discussion

We created new ultrastable recombinant SOSIP.v9 trimers that display increased thermal stability, increased purification yields and lower non-NAb reactivity compared to earlier SOSIP versions. The most thermostable trimer, SOSIP.v9.3.XL, with a melting temperature of 91.3 °C (a 14 °C increase compared to SOSIP.v6 and a 23.7 °C increase compared to SOSIP.664) induced the most potent and consistent Tier 2 autologous NAb titers after three immunizations. By characterizing the immunogenicity of a palette of SOSIP trimers with a range of melting temperatures, we found a positive correlation between thermal stability and both the magnitude and consistency of Tier 2 autologous NAb responses induced. We also used a glycan masking strategy to redirect the BG505 NAb responses from the immunodominant 241/289 glycan hole epitope to a more subdominant epitope.

It has been previously suggested that a correlation between immunogen thermostability and the potency of NAb responses exists^56^. However, this observation was based on NAb responses induced by Env trimers from diverse strains, which makes it difficult to ascertain whether the effect on the immunogenicity was due to differences in thermostability, antigenic profile, size of the glycan hole or other virus strain-specific features^57^. Here, we have demonstrated a correlation between immunogen thermostability and immunogenicity by using a palette of immunogens with different thermal stabilities derived from a single genotype (BG505). Moreover, NAb responses induced by SOSIP trimer immunogens without PNGS at 241/289 were predominantly directed to this 241/289 glycan hole, irrespective of their thermostability. Altogether, this indicates that the improvement in NAb titers is related to differences in thermal stability and not to a different antigenic profile.

Although highly thermostable immunogens from different viruses have been previously generated^35,58–60^, the mechanisms that explain the superior immunogenicity of thermally stabilized immunogens remain poorly understood. The delayed NAb responses we observed in animals vaccinated with SOSIP.v6, SOSIP.v9.4 and SOSIP.v9.4.GM, which were not detectable two weeks after the second immunization, might be a consequence of somewhat reduced epitope exposure. However, these highly stabilized immunogens may retain their native-like conformation in vivo for extended periods of time and thus increase the probability of displaying the proper antigenic determinants when they are encountered by immune cells, while less stable proteins might rapidly deteriorate into subdomains under in vivo conditions and expose epitopes that are irrelevant for neutralization. Furthermore, the rigidity provided by some stabilizing mutations reduces conformational flexibility and might help to induce neutralizing antibodies by removing entropy constraints^61^.

Previously, we found that the superior thermostability of SOSIP.v6 trimers versus earlier SOSIP forms leads to the induction of low titer heterologous neutralizing responses in some animals^35^. However, in the present study, none of the immunogens tested, including SOSIP.v6, induced any heterologous NAb responses. This is probably due to the fact that here we used a squalene emulsion-based adjuvant instead of ISCOMATRIX. Squalene emulsion is an adequate adjuvant, but it has been shown to induce less potent responses than ISCOMATRIX^62^ and thus might not have been potent enough to induce these heterologous responses.

Previous SOSIP versions have been shown to be stable for long periods of time in laboratory-controlled thermal conditions^61,63^, but ultrastable immunogens such as SOSIP.v9 trimers might be more suitable for situations in which the cold-chain conditions cannot be guaranteed during distribution and storage. Furthermore, although the induction of higher NAb titers is obviously desirable, the most important advantage of ultrastable proteins in terms of immunogenicity might be the increased consistency of the NAb responses. Based on this observation, ultrastable versions of already existing subunit vaccines could be engineered to induce more consistent coverage of the population of vaccinees and facilitate the distribution of vaccines in low-income countries.

We observed lower neutralization of the clade C Tier 1 MW965.26 viral strain in the animals vaccinated with SOSIP.v9.4 and SOSIP.v9.4.GM. These are the most thermostable trimers among the non-crosslinked immunogens we tested and include several mutations that suppress the immunodominant V3 and CD4i epitopes (316W, 306L, 308L, 201C-433C)^30–32,34^ and may result in the occlusion of other non-NAb epitopes due to reduced conformational flexibility. While the suppression of non-NAb epitopes might be beneficial for some specific applications, all evidence so far have shown that a lower induction of Abs targeting such regions is not correlated with an increased subdominant NAb response^34^. Therefore, the increased NAb titers here described for the ultrastable trimers may be attributed to their rigidity and stability rather than to the suppression of non-NAb epitopes. Recently, it has been shown that some of the antibodies elicited by HIV-1 trimer vaccines induce the degradation of the Env complex into cognate protomers^64^. Covalently linked Env proteins such as SOSIP.v9 trimers are probably less susceptible to this phenomenon, which might increase their half-life and thus improve the NAb responses elicited.

The 241/289 glycan hole BG505 Env is a highly immunodominant epitope and is targeted by immunized rabbits and, to a lesser extent, also by immunized macaques^42,45–47^. However, NAbs targeting strain-specific glycan holes are difficult to broaden^48^. Thus, NAbs targeting the BG505 241/289 glycan hole are probably only useful for neutralizing BG505 and at most a few other viruses that lack the 241 and 289 glycans. Furthermore, it has been recently shown that macaque NAbs that target the 241/289 glycan hole are less potent than those targeting other epitopes of the BG505 spike, such as the C3/V5 and V1/V3 regions^65^.

Ringe et al. previously showed that glycan masking the position 241 (241KI) of BG505 SOSIP.v4 trimers results in substantially decreased NAb responses against the parental BG505 virus, consistent with the removal of a dominant NAb epitope. Only by closing or opening specific artificial glycan holes at a different position were these BG505 SOSIP.v4 241KI trimers able to induce autologous NAb responses comparable to the ones induced by non-241KI trimers. Depending on the animal, these redirected responses targeted either the artificial glycan hole or the C3/465 epitope, or both. In contrast, we show here that glycan masking the immunodominant 241/289 epitope in the context of NAb-enhancing SOSIP.v9 trimers efficiently and consistently redirects responses to the subdominant C3/465 epitope, and possibly other epitopes, while maintaining a potent NAb response against the parental BG505 virus (Fig. 4h) and without the need of opening artificial glycan holes. Additional studies would be needed to determine if the C3/465-targeting NAb responses are likely to be broadened. In any case, the redirection capacity of filling the 241/289 hole in the context of SOSIP.v9 trimers should be useful for epitope-focusing and germline-targeting strategies that use SOSIP trimers as the platform^66,67^.

While additional studies focused on the generation of Env trimers from different genotypes without strain-specific glycan holes are necessary, it would also be crucial to immunosilence the glycan hole at the bottom of all soluble Env trimers. This region, which is not exposed in the native membrane-attached viral Env and thus generates non-NAb responses, attracts the majority of the early Ab responses targeting SOSIP trimers^68^. Several strategies, such as the incorporation of heterologous domains or additional PNGS that mask the bottom glycan hole, might further improve immunofocusing on SOSIP trimers towards bNAb epitopes.

In summary, the new SOSIP.v9 design, in particular SOSIP.v9.3.GM and SOSIP.v9.4.GM, might be a suitable platform for the generation of HIV-1 vaccine candidates. SOSIP.v9.4.GM showed higher thermostability and glycan hole immunosilencing than SOSIP.v9.3.GM. However, SOSIP.v9.4.GM produced less efficiently and showed some non-native forms. Therefore, it will be necessary to select the proper SOSIP platform depending on specific objectives or, alternatively, generate new designs that incorporate the advantages of both. Importantly, such highly thermostable and glycan masked designs might help in the enhancement and focusing of the NAb responses to specific epitopes. For instance, germline-targeting immunogens might significantly benefit from the combination of the ultrastabilization and glycan masking approaches, which might help suppress immunodominant strain-specific epitopes and redirect responses to the epitopes of interest.

## Methods

### Construct design

The BG505 SOSIP.v5 and SOSIP.v6 constructs, derived from HIV-1 subtype A BG505 *env* sequence, are extensively described and characterized elsewhere^35^. Briefly, BG505 SOSIP.v4 trimers include a set of amino acid changes to improve the expression and stability of soluble Env proteins: a TPA signal peptide (MDAMKRGLCCVLLLCGAVFVSPSQEIHARFRRGAR); 501C-605C (HxB2 numbering) (gp120-gp41 disulfide bond); 559P, 64K, and 316W trimer-stabilizing mutations; 535M and 543N, which improve trimerization; RRRRRR (R6) motif to enhance furin cleavage and a stop codon after residue 664. To further increase SOSIP proteins stability, we engineered 73C-561C intraprotomeric (SOSIP.v5) and 49C-555C interprotomeric (SOSIP.v6) disulfide bonds. The BG505 SOSIP.v9.1–9.4 constructs were generated by adding a new set of stabilizing mutations in different combinations: 1) 306L–308L mutations that stabilize the v3 loop region by hydrophobic interactions^34^; 2) MD39 mutations (304V, 319Y, 519S, 568D, 570H and 585H) to improve trimerization^33^; 3) a 201C-433C disulfide bond to increase stability and reduce V3 exposure and CD4 induction^31^. To obtain BG505 SOSIP.v9.3.GM and SOSIP.v9.4.GM glycan masked versions, PNGS motifs were introduced at positions 241 (241N) and 289 (291S), together with 240T, 271I, 288L, and 290E compensatory mutations.

Codon-optimized SOSIP.v9.1-v9.4 genes with a C-terminal His-Tag peptide (GSGSGGSGHHHHHHHH) were synthesized by Integrated DNA Technologies (Coralville, USA) and cloned by restriction-ligation into the pPPI4 vector. Point mutations to generate the glycan masked and untagged proteins were introduced by Quickchange site-directed mutagenesis (Agilent Technologies, La Jolla, CA, USA) and verified by sequencing.

### Protein expression

SOSIP proteins were expressed in transiently transfected HEK293F suspension cells (Invitrogen, cat no. R79009), maintained in FreeStyle Expression Medium (Gibco). For transfection, HIV-1 Env and furin protease-encoding plasmids mixed in a 4:1 Env to furin ratio (w/w) were incubated with PEImax (Polysciences Europe GmBH, Eppelheim, Germany) in a 3:1 (w/w) PEImax to DNA ratio and then added in the supernatant of cells at a density of 0.8–1.2 million cells/mL. Five to seven days post-transfection, supernatants were harvested, centrifuged, and filtered using Steritops (0.22 µm pore size; Millipore, Amsterdam, The Netherlands) before protein purification.

### Protein purification

Both non-crosslinked and crosslinked SOSIP proteins were purified by immunoaffinity chromatography with PGT145 or PGT151 as selecting agents, respectively^32,51^. Unpurified proteins contained in HEK293F filtered supernatants or Tris-buffered saline (TBS) solutions were captured on PGT145- or PGT151-functionalized CNBr-activated sepharose 4B beads (GE Healthcare) by overnight rolling incubation at 4 °C. Subsequently, the mixes of supernatant and beads were passed over Econo-Column chromatography columns (Biorad). The columns were then washed with three column volumes of a 0.5 M NaCl and 20 mM Tris HCl pH 8.0 solution. After elution with 3 M MgCl_2_ pH 7.5, proteins were buffer-exchanged into TN75 (75 mM NaCl, 20 mM Tris HCl pH 8.0) or PBS buffers by ultrafiltration with Vivaspin20 filters (Sartorius, Gӧttingen, Germany) of MWCO 100 kDa. Protein concentrations were determined from the A_280_ values measured on a NanoDrop2000 device (Thermo Fisher Scientific) and the molecular weight and extinction coefficient values calculated by the ProtParam Expasy webtool.

Proteins used in immunization experiments were first PGT145-purified and subsequently run through a Superdex 200 Increase 10/300 GL (GE Healthcare Life Sciences) column integrated into an NGC chromatography system (Bio-Rad). The fractions corresponding to trimers were pooled, concentrated, and filter-sterilized to avoid adverse reactions in immunized animals.

### Protein chemical crosslinking

To obtain the BG505 SOSIP.v9.3.XL protein, GLA crosslinking was performed essentially as described elsewhere^37^. Briefly, the PGT145-purified BG505 SOSIP.v9.3 protein in PBS was mixed with glutaraldehyde (GLA, Agar Scientific) to a final concentration of 7.5 mM. After a 5 min incubation at room temperature, the crosslinking reaction was stopped by the addition of 75 mM Tris buffer (pH 7.4) and the resulting protein was buffer-exchanged to TBS and further purified by PGT151 affinity chromatography, as above described.

### SDS-PAGE AND BN-PAGE

Purified proteins (2–3 µg) were run over NuPAGE 4–12% Bis-Tris and Novex Wedgewell 4–12% Tris-Glycine (both from Invitrogen) polyacrylamide gels for SDS-PAGE and BN-PAGE analysis, respectively^32^. Subsequently, gels were stained with PageBlue Protein Staining Solution (Thermo Scientific) or the Colloidal Blue Staining Kit (Life Technologies), respectively. Proteins presented in each gel were processed in parallel.

### Enzyme-linked immunosorbent assay (ELISA)

ELISA experiments with His-tagged SOSIP trimers were performed essentially as described before^13,51^. Purified proteins (1 µg/mL) were diluted in TBS and immobilized on 96-well Ni-NTA functionalized ELISA plates (Qiagen) by a 2 h incubation at room temperature. Following a double wash step with TBS to remove unbound trimers, serial dilutions of test antibodies in TBS/2% skimmed milk were added and incubated for 2 h. After 3 washes with TBS, HRP-labeled goat anti-human IgG (Jackson Immunoresearch) diluted 1:3000 in TBS/2% skimmed milk was added and incubated for 1 h, followed by 5 washes with TBS/0.05% Tween20. A developing solution (1% 3,3’,5,5’-tetramethylbenzidine (Sigma-Aldrich), 0.01% H_2_O_2_, 100 mM sodium acetate and 100 mM citric acid) allowed the colorimetric reaction, which was stopped by the addition of 0.8 M H_2_SO_4_. Finally, color development (absorption at 450 nm, OD_450_) was measured to obtain the different binding curves.

Endpoint antibody titers from rabbit sera samples were determined using Ni-NTA His-Tag-capture ELISA similarly as above described^6,51^. However, in this case, sera was diluted in TBS/2% skimmed milk/20% sheep serum and the secondary antibody was an HRP-labeled goat antirabbit IgG (Jackson Immunoresearch).

### Thermostability ELISA

For assessing the thermostability of 2G12 and PGT145 epitopes on the different SOSIP proteins, we submitted the trimers for 30 min to a set of temperatures ranging from 25 °C to 90.6 °C. Subsequently, they were diluted in TBS and immobilized on Ni-NTA as above described. Antibody binding was assessed by incubation with a single Casein Blocker (Thermo Scientific) solution with a 2G12 or PGT145 concentration close to their priorly determined IC_50_ (0.1 µg/mL). Subsequent steps to assess the binding of the test antibodies were performed as above described. Temperatures of melting (T_m_) were determined by interpolation of the temperature values that corresponded to 50% of the maximum binding signal, using Graphpad Prism 8.4.3

### Biolayer interferometry (BLI)

The BLI assay was performed using an Octet K2 (ForteBio) device at 30 °C and 1000 rpm agitation. First, test antibodies diluted in kinetics buffer (PBS/0.1% bovine serum albumin/0.02% Tween20) were loaded on protein A sensors (ForteBio) to an interference pattern shift of 1 nm. Sensors were equilibrated in kinetics buffer for 60 s to obtain a baseline prior to protein association. Subsequently, purified SOSIP trimers diluted in kinetics buffer (100 nM) were allowed to associate and dissociation for 300 s. Binding data was pre-processed and exported using the Octet software.

### Nano Differential Scanning Fluorimetry (nanoDSF)

Protein thermostability was evaluated with a Prometheus NT.48 instrument (NanoTemper Technologies). Proteins at a concentration of 1 mg/mL were loaded to the grade capillaries and the intrinsic fluorescence signal was measured while temperature was increased by 1 °C/min, with an excitation power of 40%. The temperature of onset (T_onset_) and temperature of melting (T_m_) were determined using the Prometheus NT software.

### Negative-stain electron microscopy (NS-EM)

SOSIP proteins were imaged by NS-EM essentially as described elsewhere^69^. Briefly, 3 µL protein aliquots at ∼0.03 mg/mL were applied onto a glow discharged (20 mA for 30 s) carbon-coated 400 Cu mesh grid. After 5 s, they were negatively stained with 2% (w/v) uranyl formate for 60 s. Images were taken using a FEI Tecnai T12 electron microscope operating at 120 keV.

### Site-specific glycan analysis using mass spectrometry

The BG505 SOSIP.v9.3.GM was denatured for 1 h in 50 mM Tris/HCl, pH 8.0 containing 6 M of urea and 5 mM dithiothreitol (DTT). Next, the protein was reduced and alkylated by adding 20 mM iodacetamide (IAA) and incubated for 1 h in the dark, followed by a 1 h incubation with 20 mM DTT to eliminate residual IAA. The alkylated protein was buffer-exchanged into 50 mM Tris/HCl, pH 8.0 using a Vivaspin ultrafiltration unit (Sartorius, Gӧttingen, Germany) of MWCO 3 kDa and digested separately using trypsin or chymotrypsin (Mass Spectrometry Grade, Promega) at a ratio of 1:30 (w/w) by overnight incubation. Peptides were dried, extracted using C18 Zip-tip (MerckMilipore) and dried again before being resuspended in 0.1% formic acid and analyzed by nanoLC-ESI MS with an Easy-nLC 1200 (Thermo Fisher Scientific) system coupled to a Fusion mass spectrometer (Thermo Fisher Scientific) using higher energy collision-induced dissociation (HCD) fragmentation. Peptides were separated using an EasySpray PepMap RSLC C18 column (75 µm x 75 cm). The LC conditions were as follows: 275-min linear gradient consisting of 0–32% acetonitrile in 0.1% formic acid over 240 min followed by 35 min of 80% acetonitrile in 0.1% formic acid. The flow rate was set to 200 nL/min, the spray voltage, to 2.7 kV and the temperature of the heated capillary, to 40 °C. The ion transfer tube temperature was set to 275 °C. The scan range was 400–1600 *m/z*. The HCD collision energy was set to 50%, appropriate for fragmentation of glycopeptide ions. Precursor and fragment detection were performed using an Orbitrap at a resolution MS1 = 100,000. MS2 = 30,000. The AGC target for MS1 = 4e5 and MS2 = 5e4 and injection time were MS1 = 50 and MS2 = 54.

Glycopeptide fragmentation data were extracted from the raw file using ByonicTM (Version 3.4) and ByologicTM software (Version 3.4; Protein Metrics Inc.) and evaluated manually for each glycopeptide. The peptide was scored as true-positive when the correct b and y fragment ions were observed along with oxonium ions corresponding to the glycan identified. The MS data was searched using a standard library. The precursor mass tolerance was set at 4 ppm and 10 ppm for fragments. A 1% false discovery rate (FDR) was applied. The relative amounts of each glycan at each site as well as the unoccupied proportion were determined by comparing the extracted ion chromatographic areas for different glycopeptides with an identical peptide sequence.

### Site-specific analysis of low abundance N-glycan sites using mass spectrometry

To obtain data for sites that frequently present low-intensity glycopeptide, the glycans present on the glycopeptides were homogenized to boost the intensity of these peptides. This analysis loses fine processing information but enables the ratio of oligomannose:complex:unoccupied to be determined. The remaining glycopeptides were first digested with Endo H (New England Biolabs) to deplete oligomannose- and hybrid-type glycans and leave a single GlcNAc residue at the corresponding site. The reaction mixture was then dried completely and resuspended in a mixture containing 50 mM ammonium bicarbonate and PNGase F (New England Biolabs) using only H2O18 (Sigma-Aldrich) throughout. This second reaction cleaves the remaining complex-type glycans but leaves the GlcNAc residues remaining after Endo H cleavage intact. The use of H2O18 in this reaction enables complex glycan sites to be differentiated from unoccupied glycan sites as the hydrolysis of the glycosidic bond by PNGaseF leaves a heavy oxygen isotope on the resulting aspartic acid residue. The resultant peptides were purified as outlined above and subjected to reverse-phase (RP) nanoLC-MS. Instead of the extensive N-glycan library used above, two modifications were searched for: +203 Da corresponding to a single GlcNAc, a remnant of an oligomannose/hybrid glycan, and +3 Da corresponding to the O18 deamidation product of a complex glycan. A lower HCD energy of 27% was used as glycan fragmentation was not required. Data analysis was performed as above and the relative amounts of each glycoform determined, including unoccupied peptides.

### Immunization experiment

The immunization experiment was performed by Covance Research Products Inc. (Denver, PA, USA) in female and naïve New Zealand rabbits (2.5 – 3 kg) arbitrarily distributed in 7 different groups (5 rabbits per group). Rabbits were immunized at 3 different timepoints (weeks 0, 4, and 20) with 30 µg of Env proteins diluted in PBS and formulated with Squalene emulsion (SE) adjuvant (Polymun, Klosterneuburg, Austria) in a 1:1 Env in PBS to SE ratio (v/v). Each immunization consisted of two intramuscular injections (2 × 250 µL) on both quadriceps of each animal. All immunization procedures were performed under compliance with all relevant ethical regulations for animal testing and research and received ethical approval from the Covance Institutional Animal Care and Use Committee (IACUC), with approval number C0041-19.

### Neutralization assays

TZM-bl neutralization assays were performed as described elsewhere^70^ at two different sites: Amsterdam UMC (AMC), Amsterdam, The Netherlands and Duke University Medical Center (DUMC), Durham, NC, USA. Briefly, 3-fold dilutions of heat-inactivated sera (starting form 1:20 dilution) were mixed with Env-pseudotyped viruses and incubated for 1 h at RT. Subsequently, the mix was added to TZM-bl cells seeded the previous day at a density of 17.000 cells/well. After 72 h, the cells were lysed and luciferase activity was measured using the Bright Glo Luciferase kit (Promega) in a Glomax plate reader. Neutralization titers (ID_50_ values) were determined as the serum dilution at which infectivity was inhibited by 50%.

BG505/T332N (Fig. 4h, i, Fig. S14, 17, 18) and cytoplasmatic tail truncated BG505 (BG505.dCT) (Fig. 4c–g, Fig. S11, 12, 13, 15, 16) pseudoviruses were used to measure the autologous neutralizing responses. The TZM-bl reporter cell line was obtained from John C. Kappes and Xiaoyun Wu and Tranzyme Inc. through the NIH AIDS Research and Reference Reagents Program, NIAID, NIH.

### Data representation and statistical analyses

All data representation and statistical analyses were performed using Graphpad Prism 8.4.3. Groups were compared using unpaired two-tailed Mann–Whitney U-test and multiple comparisons were analyzed with Kruskal–Wallis test, unless otherwise specified. Pearson or Spearman correlation coefficients were used to determine correlations, as specified in each case.

### Reporting summary

Further information on research design is available in the Nature Research Reporting Summary linked to this article.

## Supplementary information

Supplemental Material

Reporting Summary

## Data Availability

The data that support the findings in this study are available from the corresponding author (R.W.S.) upon reasonable request.
