# Supplementary material for: High thermostability improves neutralizing antibody responses induced by native-like HIV-1 envelope trimers
Source: NPJ Vaccines. 2022 Feb 28;7:27. doi: 10.1038/s41541-022-00446-4 (PMC8885667; doi:10.1038/s41541-022-00446-4)
Supplement: Supplementary file 1 — Supplemental Material [file 41541_2022_446_MOESM1_ESM.pdf]

```
                         ------------tPA leader-------------              49C          64K      73C
BG505 SOSIP664           MDAMKRGLCCVLLLCGAVFVSPSQEIHARFRRGARAENLWVTVYYGVPVWKDAETTLFCASDAKAYETEKHNVWATHACVPTDPNPQEIHLENVTEEFNM
BG505 SOSIPv5            MDAMKRGLCCVLLLCGAVFVSPSQEIHARFRRGARAENLWVTVYYGVPVWKDAETTLFCASDAKAYETKKHNVWATHCCVPTDPNPQEIHLENVTEEFNM
BG505 SOSIPv6            MDAMKRGLCCVLLLCGAVFVSPSQEIHARFRRGARAENLWVTVYYGVPVWKDACTTLFCASDAKAYETKKHNVWATHCCVPTDPNPQEIHLENVTEEFNM
BG505 SOSIPv9.1          MDAMKRGLCCVLLLCGAVFVSPSQEIHARFRRGARAENLWVTVYYGVPVWKDACTTLFCASDAKAYETKKHNVWATHCCVPTDPNPQEIHLENVTEEFNM
BG505 SOSIPv9.2          MDAMKRGLCCVLLLCGAVFVSPSQEIHARFRRGARAENLWVTVYYGVPVWKDACTTLFCASDAKAYETKKHNVWATHCCVPTDPNPQEIHLENVTEEFNM
BG505 SOSIPv9.3          MDAMKRGLCCVLLLCGAVFVSPSQEIHARFRRGARAENLWVTVYYGVPVWKDACTTLFCASDAKAYETKKHNVWATHCCVPTDPNPQEIHLENVTEEFNM
BG505 SOSIPv9.4          MDAMKRGLCCVLLLCGAVFVSPSQEIHARFRRGARAENLWVTVYYGVPVWKDACTTLFCASDAKAYETKKHNVWATHCCVPTDPNPQEIHLENVTEEFNM
BG505 SOSIPv9.3.GM       MDAMKRGLCCVLLLCGAVFVSPSQEIHARFRRGARAENLWVTVYYGVPVWKDACTTLFCASDAKAYETKKHNVWATHCCVPTDPNPQEIHLENVTEEFNM
BG505 SOSIPv9.4.GM       MDAMKRGLCCVLLLCGAVFVSPSQEIHARFRRGARAENLWVTVYYGVPVWKDACTTLFCASDAKAYETKKHNVWATHCCVPTDPNPQEIHLENVTEEFNM


BG505 SOSIP664           WKNNMVEQMHTDIISLWDQSLKPCVKLTPLCVTLQCTNVTNNITDDMRGELKNCSFNMTTELRDKKQKVYSLFYRLDVVQINENQGNRSNNSNKEYRLIN
BG505 SOSIPv5            WKNNMVEQMHTDIISLWDQSLKPCVKLTPLCVTLQCTNVTNNITDDMRGELKNCSFNMTTELRDKKQKVYSLFYRLDVVQINENQGNRSNNSNKEYRLIN
BG505 SOSIPv6            WKNNMVEQMHTDIISLWDQSLKPCVKLTPLCVTLQCTNVTNNITDDMRGELKNCSFNMTTELRDKKQKVYSLFYRLDVVQINENQGNRSNNSNKEYRLIN
BG505 SOSIPv9.1          WKNNMVEQMHTDIISLWDQSLKPCVKLTPLCVTLQCTNVTNNITDDMRGELKNCSFNMTTELRDKKQKVYSLFYRLDVVQINENQGNRSNNSNKEYRLIN
BG505 SOSIPv9.2          WKNNMVEQMHTDIISLWDQSLKPCVKLTPLCVTLQCTNVTNNITDDMRGELKNCSFNMTTELRDKKQKVYSLFYRLDVVQINENQGNRSNNSNKEYRLIN
BG505 SOSIPv9.3          WKNNMVEQMHTDIISLWDQSLKPCVKLTPLCVTLQCTNVTNNITDDMRGELKNCSFNMTTELRDKKQKVYSLFYRLDVVQINENQGNRSNNSNKEYRLIN
BG505 SOSIPv9.4          WKNNMVEQMHTDIISLWDQSLKPCVKLTPLCVTLQCTNVTNNITDDMRGELKNCSFNMTTELRDKKQKVYSLFYRLDVVQINENQGNRSNNSNKEYRLIN
BG505 SOSIPv9.3.GM       WKNNMVEQMHTDIISLWDQSLKPCVKLTPLCVTLQCTNVTNNITDDMRGELKNCSFNMTTELRDKKQKVYSLFYRLDVVQINENQGNRSNNSNKEYRLIN
BG505 SOSIPv9.4.GM       WKNNMVEQMHTDIISLWDQSLKPCVKLTPLCVTLQCTNVTNNITDDMRGELKNCSFNMTTELRDKKQKVYSLFYRLDVVQINENQGNRSNNSNKEYRLIN

                           201C                                 240T241N                        271I         288L290E291S
BG505 SOSIP664           CNTSAITQACPKVSFEPIPIHYCAPAGFAILKCKDKKFNGTGPCPSVSTVQCTHGIKPVVSTQLLLNGSLAEEEVMIRSENITNNAKNILVQFNTPVQIN
BG505 SOSIPv5            CNTSAITQACPKVSFEPIPIHYCAPAGFAILKCKDKKFNGTGPCPSVSTVQCTHGIKPVVSTQLLLNGSLAEEEVMIRSENITNNAKNILVQFNTPVQIN
BG505 SOSIPv6            CNTSAITQACPKVSFEPIPIHYCAPAGFAILKCKDKKFNGTGPCPSVSTVQCTHGIKPVVSTQLLLNGSLAEEEVMIRSENITNNAKNILVQFNTPVQIN
BG505 SOSIPv9.1          CNTSAITQACPKVSFEPIPIHYCAPAGFAILKCKDKKFNGTGPCPSVSTVQCTHGIKPVVSTQLLLNGSLAEEEVMIRSENITNNAKNILVQFNTPVQIN
BG505 SOSIPv9.2          CNTSAITQACPKVSFEPIPIHYCAPAGFAILKCKDKKFNGTGPCPSVSTVQCTHGIKPVVSTQLLLNGSLAEEEVMIRSENITNNAKNILVQFNTPVQIN
BG505 SOSIPv9.3          CNTSAITQACPKVSFEPIPIHYCAPAGFAILKCKDKKFNGTGPCPSVSTVQCTHGIKPVVSTQLLLNGSLAEEEVMIRSENITNNAKNILVQFNTPVQIN
BG505 SOSIPv9.4          CNTSACTQACPKVSFEPIPIHYCAPAGFAILKCKDKKFNGTGPCPSVSTVQCTHGIKPVVSTQLLLNGSLAEEEVMIRSENITNNAKNILVQFNTPVQIN
BG505 SOSIPv9.3.GM       CNTSAITQACPKVSFEPIPIHYCAPAGFAILKCKDKKFNGTGPCTNVSTVQCTHGIKPVVSTQLLLNGSLAEEEVIIRSENITNNAKNILVQLNESVQIN
BG505 SOSIPv9.4.GM       CNTSACTQACPKVSFEPIPIHYCAPAGFAILKCKDKKFNGTGPCTNVSTVQCTHGIKPVVSTQLLLNGSLAEEEVIIRSENITNNAKNILVQLNESVQIN

                           304V306L308L 316W 319Y
BG505 SOSIP664           CTRPNNNTRKSIRIGPGQAFYATGDIIGDIRQAHCNVSKATWNETLGKVVKQLRKHFGNNTIIRFANSSGGDLEVTTHSFNCGGEFFYCNTSGLFNSTWI
BG505 SOSIPv5            CTRPNNNTRKSIRIGPGQWFYATGDIIGDIRQAHCNVSKATWNETLGKVVKQLRKHFGNNTIIRFANSSGGDLEVTTHSFNCGGEFFYCNTSGLFNSTWI
BG505 SOSIPv6            CTRPNNNTRKSIRIGPGQWFYATGDIIGDIRQAHCNVSKATWNETLGKVVKQLRKHFGNNTIIRFANSSGGDLEVTTHSFNCGGEFFYCNTSGLFNSTWI
BG505 SOSIPv9.1          CTRPNNNTRKLILIGPGQWFYATGDIIGDIRQAHCNVSKATWNETLGKVVKQLRKHFGNNTIIRFANSSGGDLEVTTHSFNCGGEFFYCNTSGLFNSTWI
BG505 SOSIPv9.2          CTRPNNNTVKSIRIGPGQAFYYTGDIIGDIRQAHCNVSKATWNETLGKVVKQLRKHFGNNTIIRFANSSGGDLEVTTHSFNCGGEFFYCNTSGLFNSTWI
BG505 SOSIPv9.3          CTRPNNNTVKSIRIGPGQWFYYTGDIIGDIRQAHCNVSKATWNETLGKVVKQLRKHFGNNTIIRFANSSGGDLEVTTHSFNCGGEFFYCNTSGLFNSTWI
BG505 SOSIPv9.4          CTRPNNNTRKLILIGPGQWFYATGDIIGDIRQAHCNVSKATWNETLGKVVKQLRKHFGNNTIIRFANSSGGDLEVTTHSFNCGGEFFYCNTSGLFNSTWI
BG505 SOSIPv9.3.GM       CTRPNNNTVKSIRIGPGQWFYYTGDIIGDIRQAHCNVSKATWNETLGKVVKQLRKHFGNNTIIRFANSSGGDLEVTTHSFNCGGEFFYCNTSGLFNSTWI
BG505 SOSIPv9.4.GM       CTRPNNNTRKLILIGPGQWFYATGDIIGDIRQAHCNVSKATWNETLGKVVKQLRKHFGNNTIIRFANSSGGDLEVTTHSFNCGGEFFYCNTSGLFNSTWI

                                                              433C
BG505 SOSIP664           SNTSVQGSNSTGSNDSITLPCRIKQIINMWQRIGQAMYAPPIQGVIRCVSNITGLILTRDGGSTNSTTETFRPGGGDMRDNWRSELYKYKVVKIEPLGVA
BG505 SOSIPv5            SNTSVQGSNSTGSNDSITLPCRIKQIINMWQRIGQAMYAPPIQGVIRCVSNITGLILTRDGGSTNSTTETFRPGGGDMRDNWRSELYKYKVVKIEPLGVA
BG505 SOSIPv6            SNTSVQGSNSTGSNDSITLPCRIKQIINMWQRIGQAMYAPPIQGVIRCVSNITGLILTRDGGSTNSTTETFRPGGGDMRDNWRSELYKYKVVKIEPLGVA
BG505 SOSIPv9.1          SNTSVQGSNSTGSNDSITLPCRIKQIINMWQRIGQAMYAPPIQGVIRCVSNITGLILTRDGGSTNSTTETFRPGGGDMRDNWRSELYKYKVVKIEPLGVA
BG505 SOSIPv9.2          SNTSVQGSNSTGSNDSITLPCRIKQIINMWQRIGQAMYAPPIQGVIRCVSNITGLILTRDGGSTNSTTETFRPGGGDMRDNWRSELYKYKVVKIEPLGVA
BG505 SOSIPv9.3          SNTSVQGSNSTGSNDSITLPCRIKQIINMWQRIGQAMYAPPIQGVIRCVSNITGLILTRDGGSTNSTTETFRPGGGDMRDNWRSELYKYKVVKIEPLGVA
BG505 SOSIPv9.4          SNTSVQGSNSTGSNDSITLPCRIKQIINMWQRIGQCMYAPPIQGVIRCVSNITGLILTRDGGSTNSTTETFRPGGGDMRDNWRSELYKYKVVKIEPLGVA
BG505 SOSIPv9.3.GM       SNTSVQGSNSTGSNDSITLPCRIKQIINMWQRIGQAMYAPPIQGVIRCVSNITGLILTRDGGSTNSTTETFRPGGGDMRDNWRSELYKYKVVKIEPLGVA
BG505 SOSIPv9.4.GM       SNTSVQGSNSTGSNDSITLPCRIKQIINMWQRIGQCMYAPPIQGVIRCVSNITGLILTRDGGSTNSTTETFRPGGGDMRDNWRSELYKYKVVKIEPLGVA

                          501C                519S            535M   543N     555C 559P 561C 568D570H        585H
BG505 SOSIP664           PTRCKRRVVGRRRRRRAVGIGAVFLGFLGAAGSTMGAASMTLTVQARNLLSGIVQQQSNLLRAPEAQQHLLKLTVWGIKQLQARVLAVERYLRDQQLLGI
BG505 SOSIPv5            PTRCKRRVVGRRRRRRAVGIGAVFLGFLGAAGSTMGAASMTLTVQARNLLSGIVQQQSNLLRAPECQQHLLKLTVWGIKQLQARVLAVERYLRDQQLLGI
BG505 SOSIPv6            PTRCKRRVVGRRRRRRAVGIGAVFLGFLGAAGSTMGAASMTLTVQARNLLSGIVQQQSNCLRAPECQQHLLKLTVWGIKQLQARVLAVERYLRDQQLLGI
BG505 SOSIPv9.1          PTRCKRRVVGRRRRRRAVGIGAVSLGFLGAAGSTMGAASMTLTVQARNLLSGIVQQQSNCLRAPECQQHLLKDTHWGIKQLQARVLAVEHYLRDQQLLGI
BG505 SOSIPv9.2          PTRCKRRVVGRRRRRRAVGIGAVSLGFLGAAGSTMGAASMTLTVQARNLLSGIVQQQSNCLRAPECQQHLLKDTHWGIKQLQARVLAVEHYLRDQQLLGI
BG505 SOSIPv9.3          PTRCKRRVVGRRRRRRAVGIGAVSLGFLGAAGSTMGAASMTLTVQARNLLSGIVQQQSNCLRAPECQQHLLKDTHWGIKQLQARVLAVEHYLRDQQLLGI
BG505 SOSIPv9.4          PTRCKRRVVGRRRRRRAVGIGAVSLGFLGAAGSTMGAASMTLTVQARNLLSGIVQQQSNCLRAPECQQHLLKDTHWGIKQLQARVLAVEHYLRDQQLLGI
BG505 SOSIPv9.3.GM       PTRCKRRVVGRRRRRRAVGIGAVSLGFLGAAGSTMGAASMTLTVQARNLLSGIVQQQSNCLRAPECQQHLLKDTHWGIKQLQARVLAVEHYLRDQQLLGI
BG505 SOSIPv9.4.GM       PTRCKRRVVGRRRRRRAVGIGAVSLGFLGAAGSTMGAASMTLTVQARNLLSGIVQQQSNCLRAPECQQHLLKDTHWGIKQLQARVLAVEHYLRDQQLLGI

                               605C
BG505 SOSIP664           WGCSGKLICCTNVPWNSSWSNRNLSEIWDNMTWLQWDKEISNYTQIIYGLLEESQNQQEKNEQDLLALD*
BG505 SOSIPv5            WGCSGKLICCTNVPWNSSWSNRNLSEIWDNMTWLQWDKEISNYTQIIYGLLEESQNQQEKNEQDLLALD*
BG505 SOSIPv6            WGCSGKLICCTNVPWNSSWSNRNLSEIWDNMTWLQWDKEISNYTQIIYGLLEESQNQQEKNEQDLLALD*
BG505 SOSIPv9.1          WGCSGKLICCTNVPWNSSWSNRNLSEIWDNMTWLQWDKEISNYTQIIYGLLEESQNQQEKNEQDLLALD*
BG505 SOSIPv9.2          WGCSGKLICCTNVPWNSSWSNRNLSEIWDNMTWLQWDKEISNYTQIIYGLLEESQNQQEKNEQDLLALD*
BG505 SOSIPv9.3          WGCSGKLICCTNVPWNSSWSNRNLSEIWDNMTWLQWDKEISNYTQIIYGLLEESQNQQEKNEQDLLALD*
BG505 SOSIPv9.4          WGCSGKLICCTNVPWNSSWSNRNLSEIWDNMTWLQWDKEISNYTQIIYGLLEESQNQQEKNEQDLLALD*
BG505 SOSIPv9.3.GM       WGCSGKLICCTNVPWNSSWSNRNLSEIWDNMTWLQWDKEISNYTQIIYGLLEESQNQQEKNEQDLLALD*
BG505 SOSIPv9.4.GM       WGCSGKLICCTNVPWNSSWSNRNLSEIWDNMTWLQWDKEISNYTQIIYGLLEESQNQQEKNEQDLLALD*
```

Supplementary Figure 1. Amino acid sequence alignment of ultrastable SOSIP.v9 trimers and their SOSIP predecessors. The alignment includes previous versions of BG505 SOSIP (SOSIP.664, SOSIP.v5 and SOSIP.v6), the ultrastable (BG505 SOSIP.v9.1-v9.4) and the glycan hole-masked (BG505 SOSIPv9.3.GM and BG505 SOSIPv9.4.GM) proteins. SOSIP and trimer-stabilizing mutations are indicated in bold; mutations that result in extra disulfide bonds, in red; stabilizing mutations introduced to generate the new v9.1-v9.4 SOSIP trimers, in green; and mutations to knock-in the 241 and 289 glycans, in purple (HxB2 numbering).

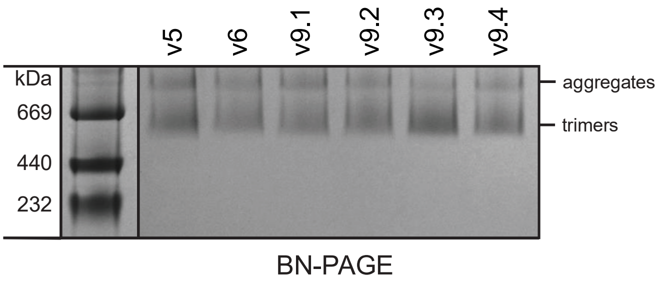

Supplementary Figure 2. BN-PAGE analysis of PGT145-purified SOSIP.v9 and SOSIP.v5 and SOSIP.v6 reference proteins.

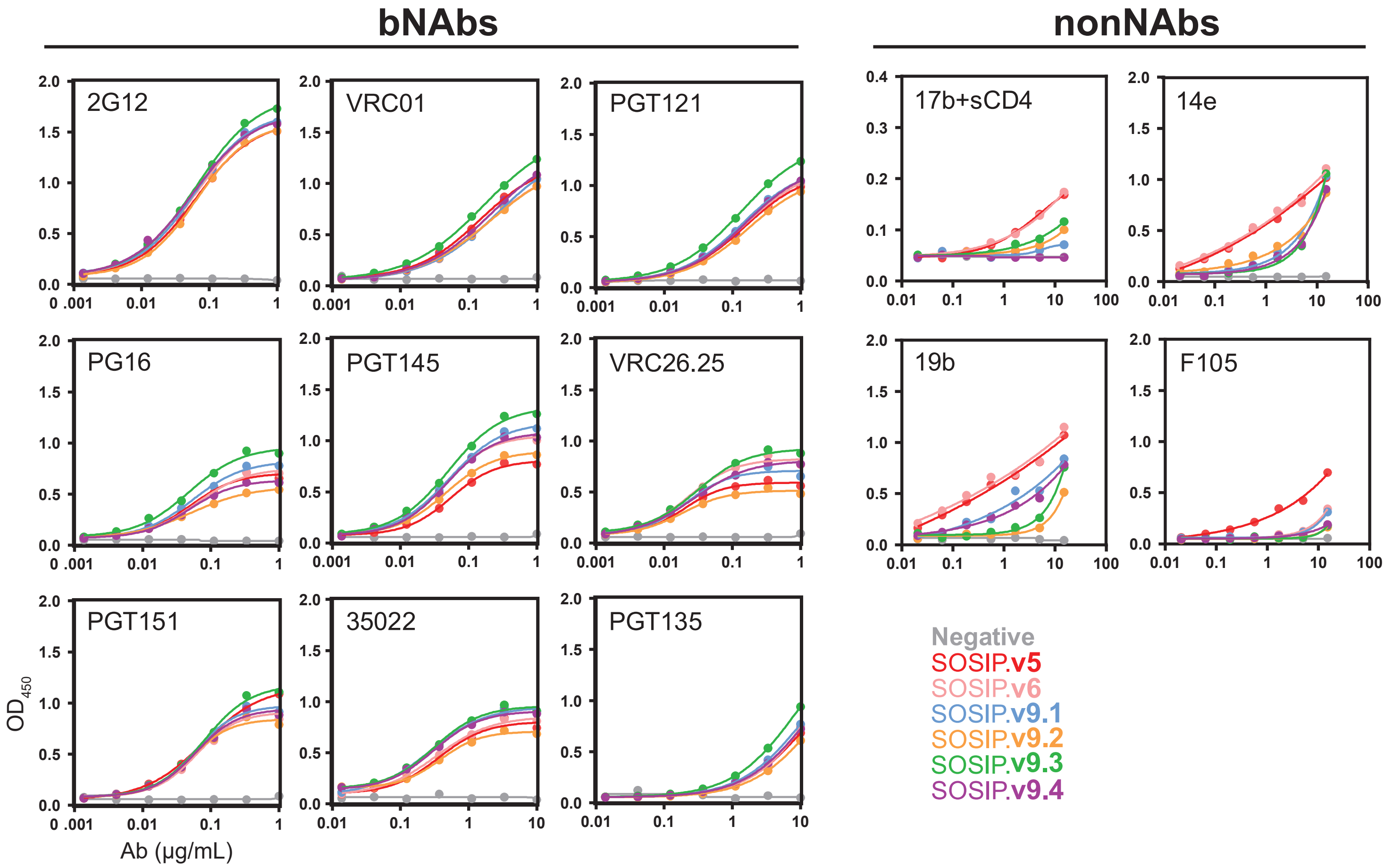

Supplementary Figure 3. Ni-NTA-capture enzyme-linked immunosorbent assay with PGT145-purified SOSIP.v9 and the SOSIP.v5 and SOSIP.v6 control proteins against a panel of bNAbs and non-NAbs. Binding curves of one of the duplicates used to calculate the area under the curve (AUC) values represented in Figure 1E.

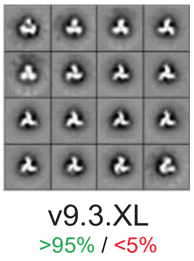

Supplementary Figure 4. Negative-stain electron microscopy 2D class average representation of the chemically crosslinked SOSIP.v9.3.XL protein. The percentages of native-like trimers and non-native-like trimers are shown in green and red, respectively.

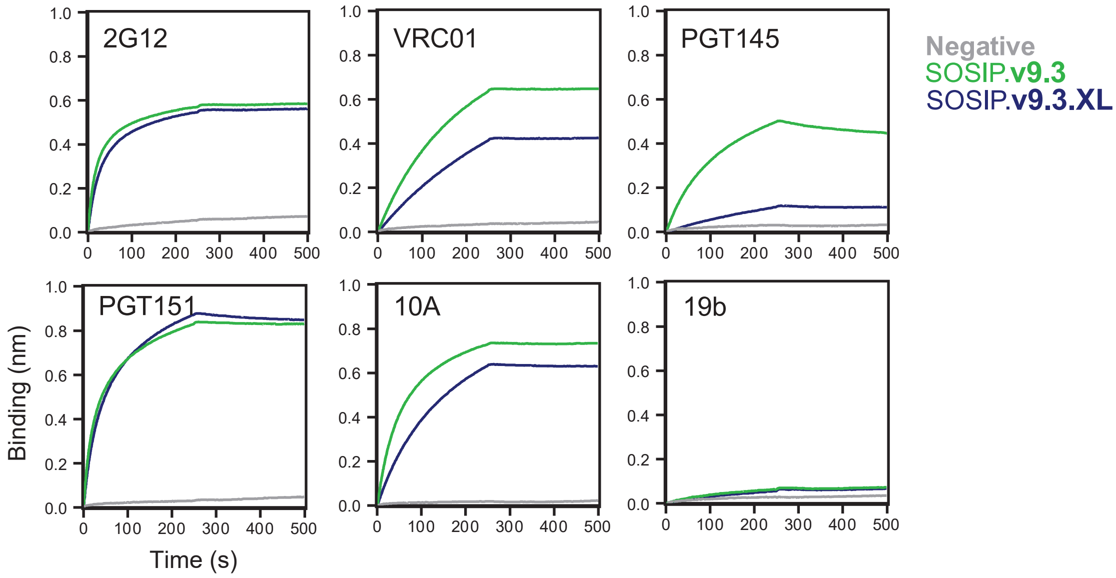

Supplementary Figure 5. Protein A bio-layer interferometry assay with SOSIP.v9.3 and SOSIP.v9.3.XL untagged proteins against a small panel or relevant mAbs. Proteins were purified by PGT145-affinity chromatography and chemical crosslinking and PGT151-affinity chromatography, respectively, as described in the methods section. The assay was performed in duplicate and the curves presented correspond to one of these experiments.

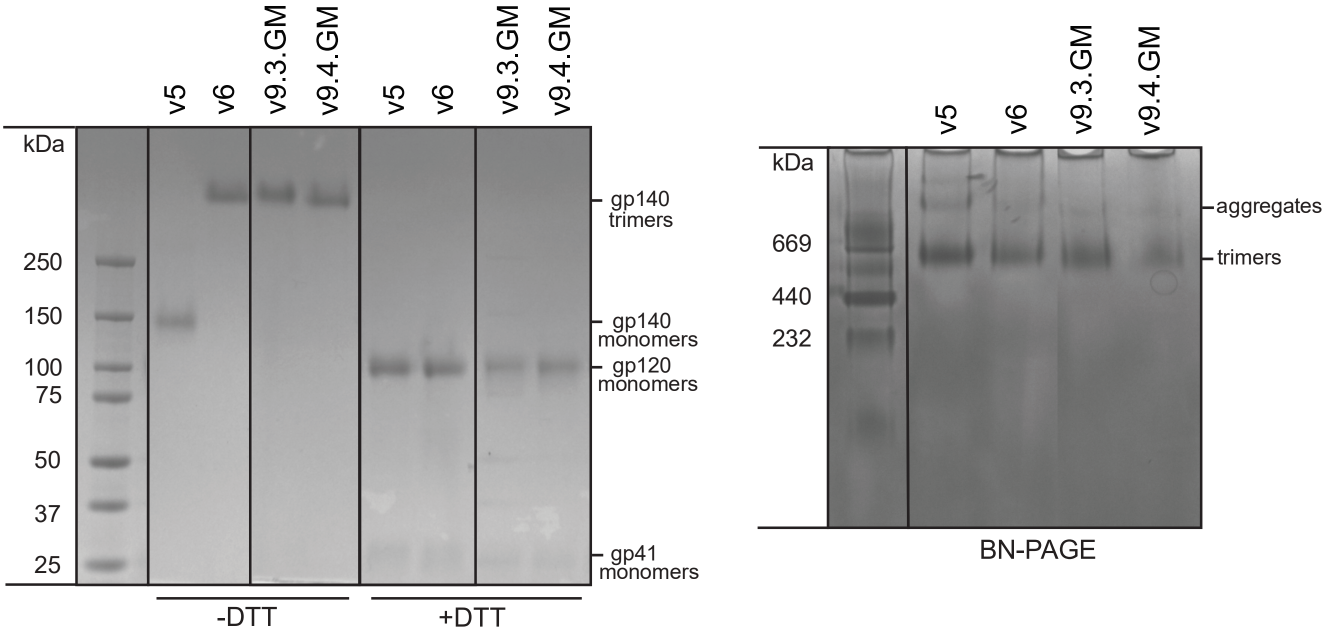

Supplementary Figure 6. SDS-PAGE and BN-PAGE analysis of non-GM and GM versions of SOSIP.v9.3 and SOSIP.v9.4 trimers. SDS-PAGE analysis was performed both in non-reducing (-DTT) and reducing (+DTT) conditions.

	N88	N133	N142	N156	N160	N185e	N185h	N197	N234	N241	N262	N276	N289	N295	N301	N332	N339	N355	N363	N386	N392	N398	N406	N411	N448	N462	N611	N618	N625	N637
Occupancy	96	88	65	100	100	97	100	95	95	90	98	100	66	100	70	100	88	100	100	100	ND	100	100	100	100	100	84	100	65	94
High Mannose	30	75	51	100	100	5	0	61	94	88	98	75	66	100	70	100	88	35	100	100	ND	0	0	100	97	1	14	0	65	49
M9	0			0	15	0		4			55	0				72	62	0	59	86				0	66	0	0			0
M8	0			45	60	0		3			33	25				22	22	1	27	14				0	31	0	4			4
M7	1			31	22	1		14			5	29				2	2	4	7	0				16	0	0	3			7
M6	6			11	3	0		8			3	0				1	1	5	8	0				24	0	0	2			5
M5	14			8	0	3		20			2	0				3	0	13	0	0				59	0	1	4			12
M4	0			4	0	0		0			0	2				0	0	1	0	0				0	0	0	0			1
M3	0			0	0	0		0			0	0				0	0	0	0	0				0	0	0	0			0
Hybrid	6			0	0	0		6			0	20				0	0	4	0	0				0	0	0	1			8
Fhybrid	2			0	0	0		6			0	0				0	0	7	0	0				0	0	1	0			11
Complex	66	12	14	0	0	91	100	32	0	2	0	24	0	0	0	0	0	64	0	0	ND	100	100	0	3	98	69	99	0	45
A1	6			0	0	0		1				0						0								0	1	0		3
FA1	1			0	0	3		6				0						9								2	2	0		8
A2/A1B	19			0	0	0		1				8						0								2	0	0		1
FA2/FA1B	16			0	0	49		20				5						29								60	9	39		26
A3/A2B	7			0	0	0		0				6						0								0	0	0		0
FA3/FA2B	16			0	0	36		6				5						24								32	47	52		7
A4/A3B	0			0	0	0		0				0						0								0	0	0		0
FA4/FA3B	2			0	0	3		0				0						2								2	10	8		0
Unoccupied	4	12	35	0	0	3	0	5	5	10	2	0	34	0	30	0	12	0	0	0	ND	0	0	0	0	0	16	0	35	6

Supplementary Figure 7. Tabulated values of the site-specific glycan analysis of the SOSIP.v9.3.GM protein.

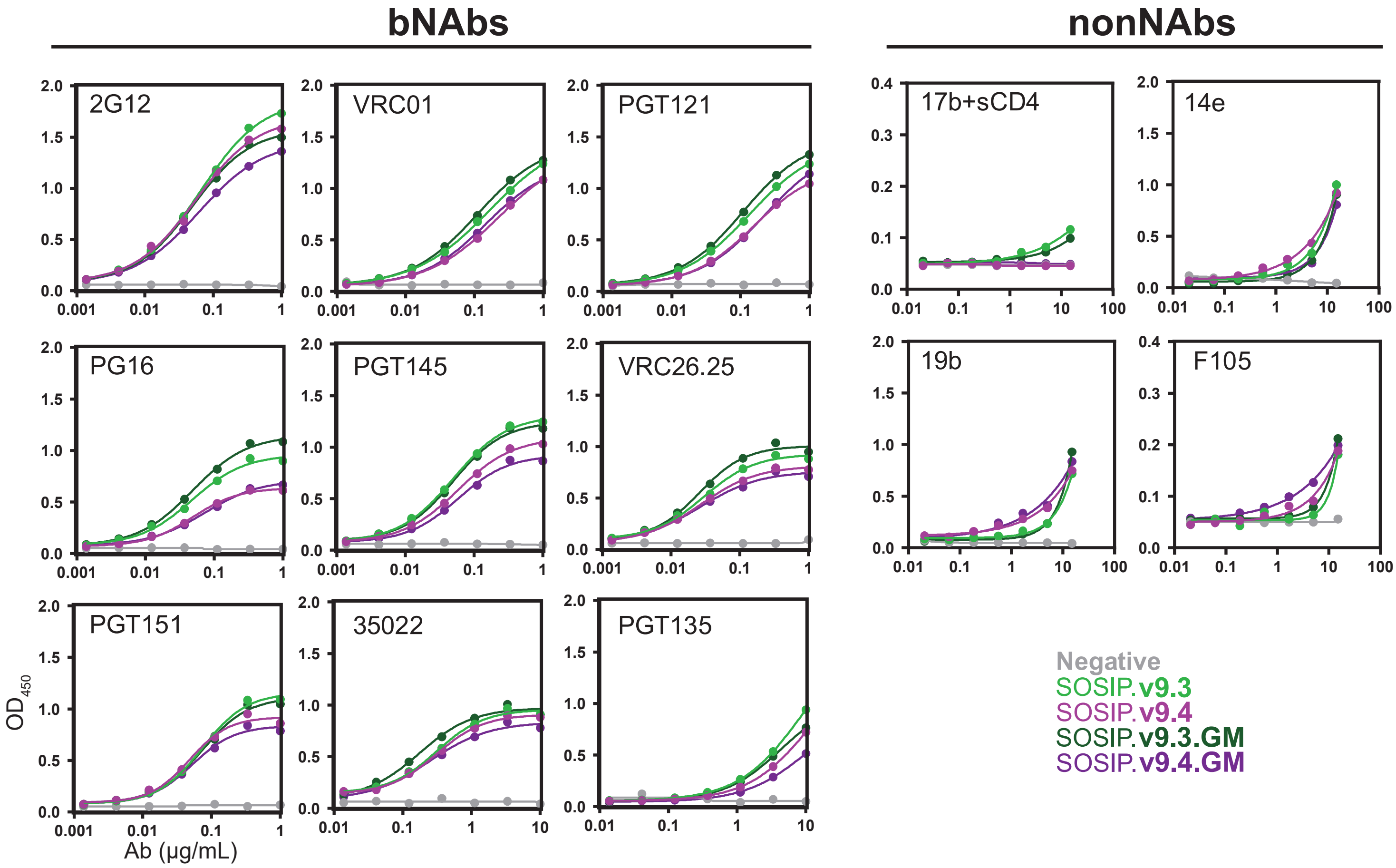

Supplementary Figure 8. Ni-NTA-capture enzyme-linked immunosorbent assay with PGT145-purified non-GM and GM versions of SOSIP.v9.3 and SOSIP.v9.4 trimers against a panel of bNAbs and non-NAbs. Binding curves of one of the two duplicate experiments performed.

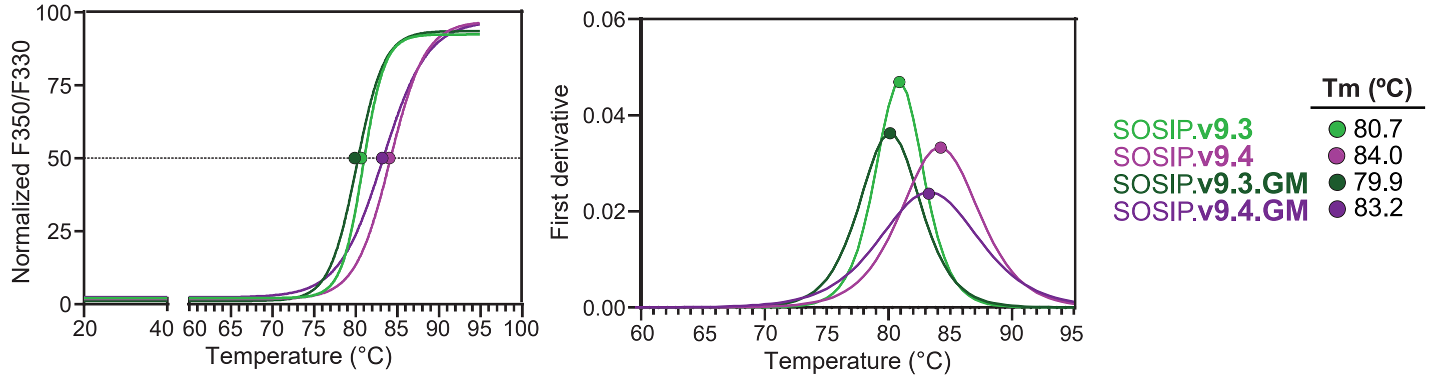

Supplementary Figure 9. Thermostability of the non-GM and GM versions of SOSIP.v9.3 and SOSIP.v9.4 proteins. a Fluorescence ratio (F350/F330) (left) and first derivative of the signal (right) data determined by nanoDSF and used to calculate the temperature of melting (Tm) values of PGT145-purifed non-GM and GM SOSIP.v9.3 and SOSIP.v9.4 proteins. Dots represent the Tm value of each protein, calculated as the temperature at which the signal is half of the maximum signal (left) or the value of the first derivative is maximized (right). b Temperatures of melting of each protein, measured by nanoDSF.

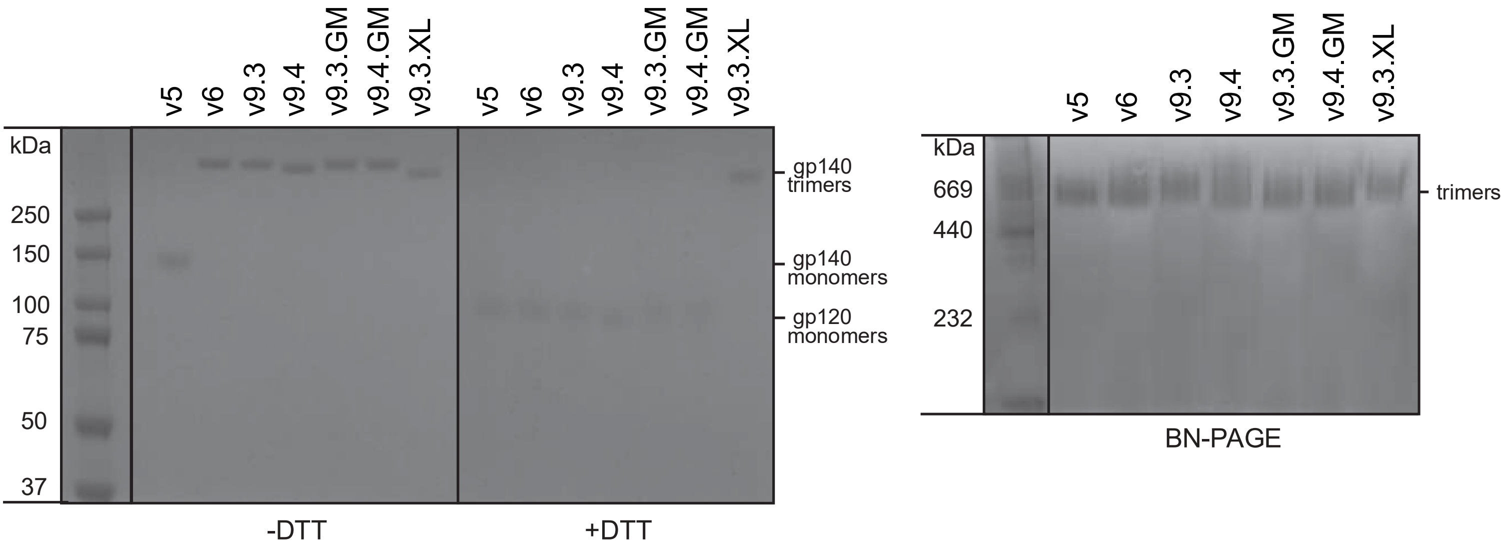

Supplementary Figure 10. SDS-PAGE and BN-PAGE analysis of proteins used in the immunization experiment. Untagged proteins for immunization were purified by PGT145- or PGT151-affinity chromatography and a subsequent step of size exclusion chromatography to remove aggregates. SDS-PAGE analysis was performed both in non-reducing (-DTT) and reducing (+DTT) conditions.

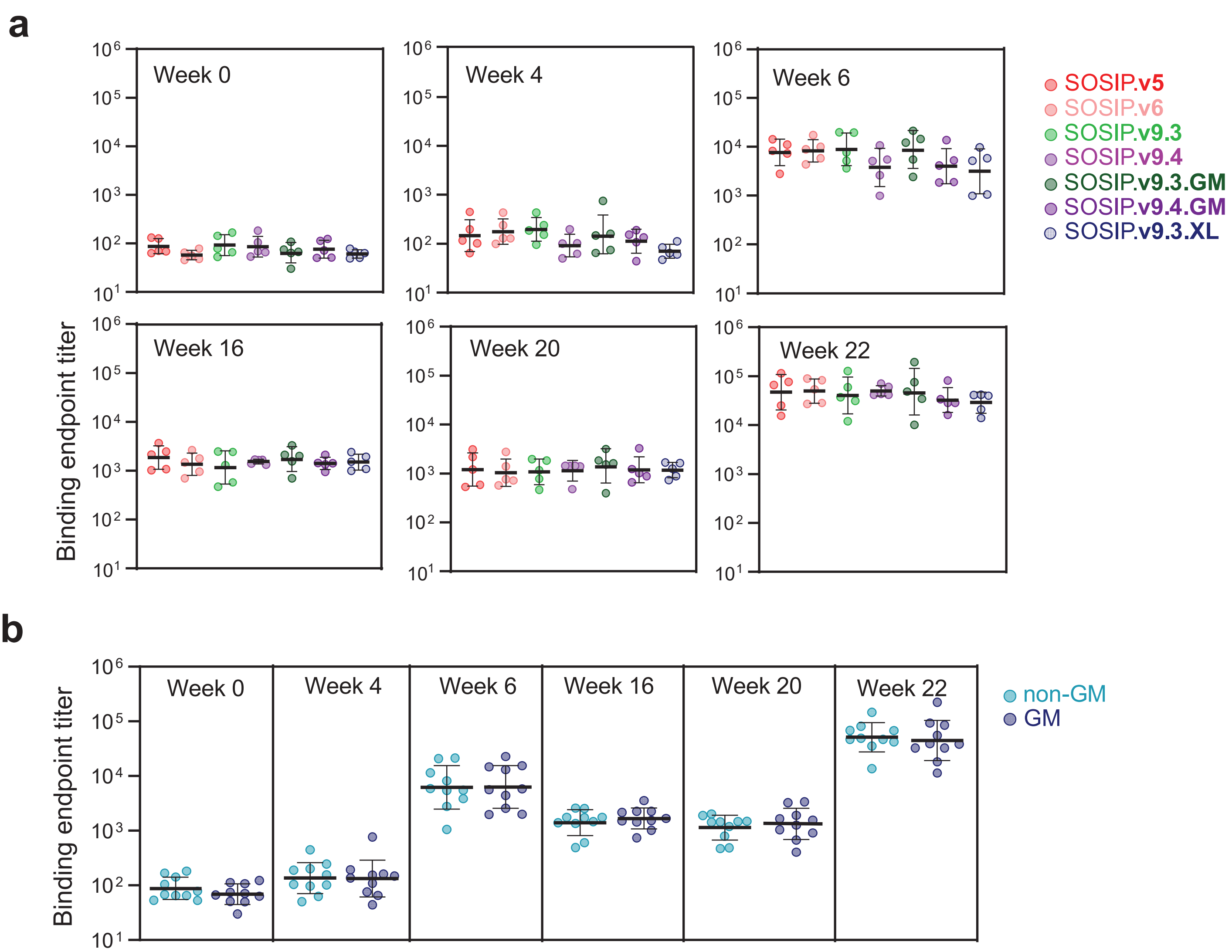

Supplementary Figure 11. Antibody-binding titers over time against the autologous Env protein. a Endpoint antibody-binding titers over time against BG505 SOSIP.v5 trimer as measured by Ni-NTA enzyme-linked immunosorbent assay. The individual values, mean binding titers and standard deviation of each group are represented. No significant differences were found by **a** Kruskal-Wallis statistical test between groups at any timepoint tested. **b** Comparison of endpoint antibody-binding titers elicited in animals vaccinated with SOSIP.v9.3 and SOSIP.v9.4 non-glycan masked (non-GM) immunogens versus animals vaccinated with SOSIP.v9.3 and SOSIP.v9.4 glycan masked (GM) immunogens. Dots represent titer values for each animal, while the line and error bars correspond to the geometric mean and geometric standard deviation of the values in each group. No significant differences were found by a Mann-Whitney statistical test between groups at any timepoint tested.

			BG505 dCT					
			Tier 2					
			Clade A					
		Site	AMC					
Group	Immunogen	Rabbit ID	Wk0	Wk4	Wk6	Wk16	Wk20	Wk22
1	SOSIP.v5	UA129	<20	<20	77	309	164	2139
		UA130	<20	<20	155	<20	<20	<20
		UA131	<20	<20	<20	<20	20	177
		UA132	<20	<20	413	39	82	3121
		UA133	<20	<20	1165	798	<20	5813
2	SOSIP.v6	UA134	<20	<20	<20	<20	239	58
		UA135	<20	<20	<20	71	217	4217
		UA136	<20	<20	<20	<20	58	487
		UA137	<20	<20	<20	<20	<20	2418
		UA138	<20	<20	62	31	<20	1402
3	SOSIP.v9.3	UA139	<20	<20	120	79	69	213
		UA140	<20	<20	81	128	94	4557
		UA141	<20	<20	<20	<20	<20	309
		UA142	<20	<20	837	440	346	10848
		UA143	<20	<20	<20	<20	48	345
4	SOSIP.v9.4	UA144	<20	<20	<20	40	51	393
		UA145	<20	<20	<20	246	320	2080
		UA146	<20	<20	<20	78	283	3155
		UA147	<20	<20	<20	<20	<20	735
		UA148	<20	<20	<20	60	202	1840
5	SOSIP.v9.3.XL	UA149	<20	<20	<20	64	89	4765
		UA150	<20	<20	215	25	58	2206
		UA151	<20	<20	<20	<20	420	3351
		UA152	<20	<20	60	152	351	4313
		UA153	<20	<20	29	191	270	4682
6	SOSIP.v9.3.GM	UA154	<20	<20	<20	<20	<20	133
		UA155	<20	<20	694	383	347	9647
		UA156	<20	<20	<20	<20	<20	<20
		UA157	<20	<20	41	<20	<20	588
		UA158	<20	<20	<20	83	38	2043
7	SOSIP.v9.4.GM	UA159	<20	<20	<20	42	94	1230
		UA160	<20	<20	<20	109	122	1228
		UA161	<20	<20	<20	20	<20	1133
		UA162	<20	<20	<20	91	292	793
		UA163	<20	<20	<20	145	26	4392

Neutralization titers (ID50)	
(light grey)	20-40
(yellow)	41-100
(orange)	101-1000
(red)	>1001

Supplementary Figure 12. Midpoint neutralization titers (ID50) over time for sera of the immunized rabbits against a virus pseudotyped with a cytoplasmatic tail truncated BG505 Env (BG505.dCT). ID50 values were determined in a TZM-bl neutralization assay as described in the methods section.

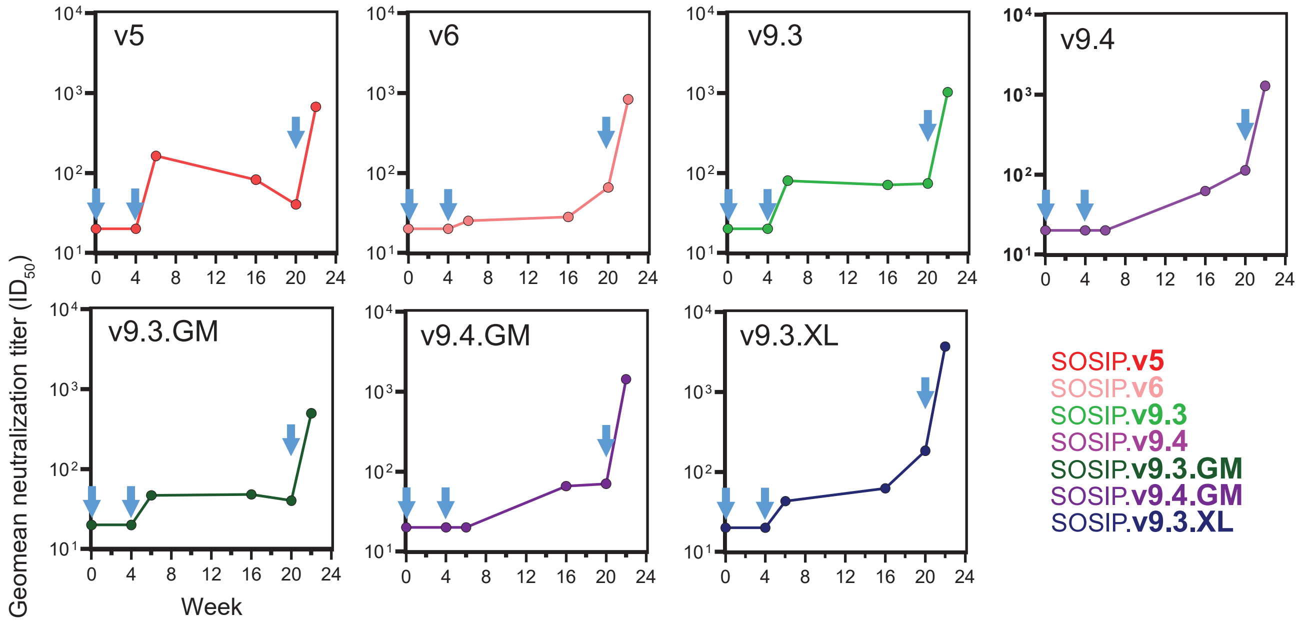

Supplementary Figure 13. Midpoint neutralization titers (ID50) over time for sera of the immunized rabbits against a BG505.dCT virus. A set of graphs shows the evolution of the geometric mean neutralization titers for each group individually. ID50 values are presented on Figure S12.

			MLV		SF162	MW965.26	BG505/T332N		92UG020	REJO	SHIVp3	TRO.11	Ce1176_A3		25710-2.43		BJOX002000.03.2	X1632-S2-B10	246-G3_C10_2	CH119.10	Ce703010217_B6	CNE55
			Neg. control		Tier 1A	Tier 1A	Tier 2		Tier 2	Tier 2	Tier 2	Tier 2	Tier 2		Tier 2		Tier 2	Tier 2	Tier 2	Tier 2	Tier 2	Tier 2
			-		Clade B	Clade C	Clade A		Clade A	Clade B	Clade B	Clade B	Clade C		Clade C		CRF07_BC	Clade G	Clade AC	CRF07_BC	Clade C	CRF01_AE
Group	Immunogen	Rabbit ID	AMC	DUMC	AMC	DUMC	AMC	DUMC	AMC	AMC	AMC	DUMC	AMC	DUMC	AMC	DUMC	DUMC	DUMC	DUMC	DUMC	DUMC	DUMC
1	SOSIP.v5	UA129	20	29	36	581	1531	1691	78	22	96	44	<20	22	108	25	<20	<20	30	38	<20	<20
		UA130	<20	27	<20	78	<20	33	<20	<20	<20	38	<20	23	<20	<20	<20	<20	25	22	<20	<20
		UA131	<20	26	<20	422	258	108	<20	<20	20	33	<20	27	<20	<20	<20	<20	38	29	<20	<20
		UA132	<20	34	37	1215	2023	800	<20	<20	<20	52	<20	33	<20	28	27	<20	39	33	<20	<20
		UA133	<20	27	<20	724	5688	2966	<20	<20	<20	34	<20	31	<20	34	35	33	49	33	<20	29
2	SOSIP.v6	UA134	<20	30	97	1384	<20	93	<20	<20	<20	44	<20	30	<20	32	28	28	38	34	<20	<20
		UA135	<20	<20	<20	98	831	872	<20	<20	<20	25	<20	<20	<20	<20	<20	<20	<20	<20	<20	<20
		UA136	<20	28	<20	66	112	217	<20	<20	58	23	<20	<20	<20	<20	<20	<20	22	<20	<20	<20
		UA137	<20	37	<20	471	419	624	<20	<20	<20	65	<20	44	<20	40	37	36	45	32	<20	28
		UA138	<20	<20	40	2063	795	<20	63	<20	<20	23	<20	<20	<20	29	28	<20	27	53	<20	25
3	SOSIP.v9.3	UA139	38	98	<20	180	30	188	315	44	142	122	42	93	99	95	71	67	75	92	59	61
		UA140	<20	30	20	60	1334	1648	20	<20	20	40	<20	24	<20	28	<20	<20	32	24	<20	<20
		UA141	<20	<20	<20	63	92	120	<20	<20	<20	<20	<20	<20	<20	<20	<20	<20	<20	<20	<20	<20
		UA142	<20	<20	<20	163	14704	7474	23	<20	<20	<20	<20	<20	<20	<20	<20	<20	<20	<20	<20	<20
		UA143	<20	26	<20	284	127	173	<20	<20	<20	28	<20	<20	<20	33	26	<20	37	30	<20	<20
4	SOSIP.v9.4	UA144	<20	<20	<20	<20	196	267	<20	<20	<20	<20	<20	<20	<20	<20	<20	<20	<20	<20	<20	<20
		UA145	<20	<20	<20	44	763	675	<20	<20	<20	<20	<20	<20	<20	<20	<20	<20	<20	<20	<20	<20
		UA146	<20	<20	<20	25	435	747	<20	<20	<20	29	<20	<20	<20	28	23	<20	30	<20	<20	<20
		UA147	<20	<20	<20	<20	199	166	<20	<20	<20	<20	<20	<20	<20	<20	<20	<20	<20	<20	<20	<20
		UA148	<20	<20	<20	<20	1024	1269	<20	<20	<20	<20	<20	<20	<20	37	<20	<20	28	<20	<20	<20
5	SOSIP.v9.3.XL	UA149	<20	41	<20	123	1344	962	<20	<20	<20	86	<20	49	<20	55	44	40	81	39	24	26
		UA150	<20	37	<20	206	1191	590	<20	<20	<20	54	<20	41	<20	49	42	38	53	38	32	28
		UA151	<20	<20	<20	146	283	554	<20	<20	44	<20	<20	<20	<20	<20	<20	<20	<20	<20	<20	<20
		UA152	<20	<20	<20	54	2157	1247	<20	<20	20	27	<20	<20	<20	<20	<20	<20	22	<20	<20	<20
		UA153	<20	<20	<20	36	1515	1470	<20	<20	<20	22	<20	<20	<20	27	24	22	36	37	26	<20
6	SOSIP.v9.3.GM	UA154	<20	<20	<20	209	48	82	<20	<20	<20	37	<20	20	<20	26	<20	<20	35	<20	<20	<20
		UA155	<20	36	<20	119	7555	6520	<20	<20	<20	55	<20	40	<20	36	35	29	50	<20	<20	<20
		UA156	<20	<20	<20	32	<20	35	<20	<20	<20	28	<20	<20	<20	<20	<20	<20	24	<20	<20	<20
		UA157	<20	<20	<20	1292	237	261	<20	<20	<20	27	<20	<20	<20	26	<20	<20	39	25	24	<20
		UA158	<20	<20	21	30	1063	1016	71	<20	24	<20	<20	<20	<20	<20	<20	<20	<20	<20	<20	<20
7	SOSIP.v9.4.GM	UA159	<20	26	<20	29	1307	668	20	<20	<20	40	<20	29	<20	34	24	<20	40	29	<20	<20
		UA160	<20	<20	<20	22	704	593	<20	25	<20	33	<20	<20	<20	<20	<20	<20	29	30	<20	<20
		UA161	<20	<20	<20	<20	695	605	<20	<20	<20	<20	<20	<20	<20	<20	<20	<20	34	28	<20	<20
		UA162	<20	<20	<20	<20	704	437	<20	<20	<20	<20	<20	<20	<20	<20	<20	<20	<20	<20	<20	<20
		UA163	<20	22	<20	82	2247	1930	<20	<20	<20	33	<20	<20	<20	36	22	<20	37	49	<20	<20

Neutralization titers (ID50)
20-40
41-100
101-1000
>1001

Supplementary Figure 14. Midpoint neutralization titers (ID50) for week 22 sera of the immunized rabbits against a panel of viruses pseudotyped with different Tier 1 and Tier 2 Env proteins, including the BG505/T332N autologous virus. ID50 values were determined in a TZM-bl neutralization assay as described in the methods section in two different locations: Academic Medical Center (AMC) and Duke University Medical Center (DUMC).

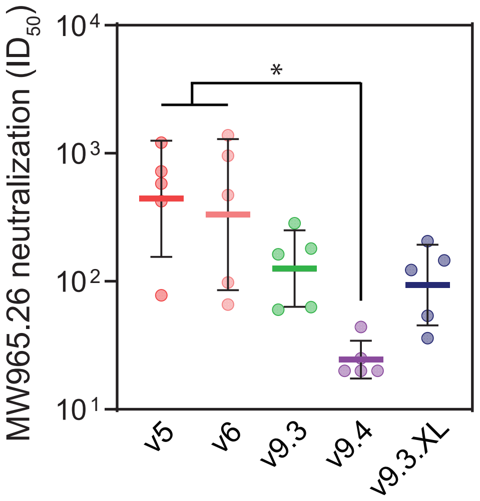

Supplementary Figure 15. Midpoint neutralization titers (ID50) for week 22 sera of the immunized rabbits against a Tier 1A MW965.25 pseudovirus. ID50 values are presented on Figure S14. Individual ID50 values, geometric means and geometric standard deviations are represented. A Kruskal-Wallis statistical test indicates significant differences (*) between groups.

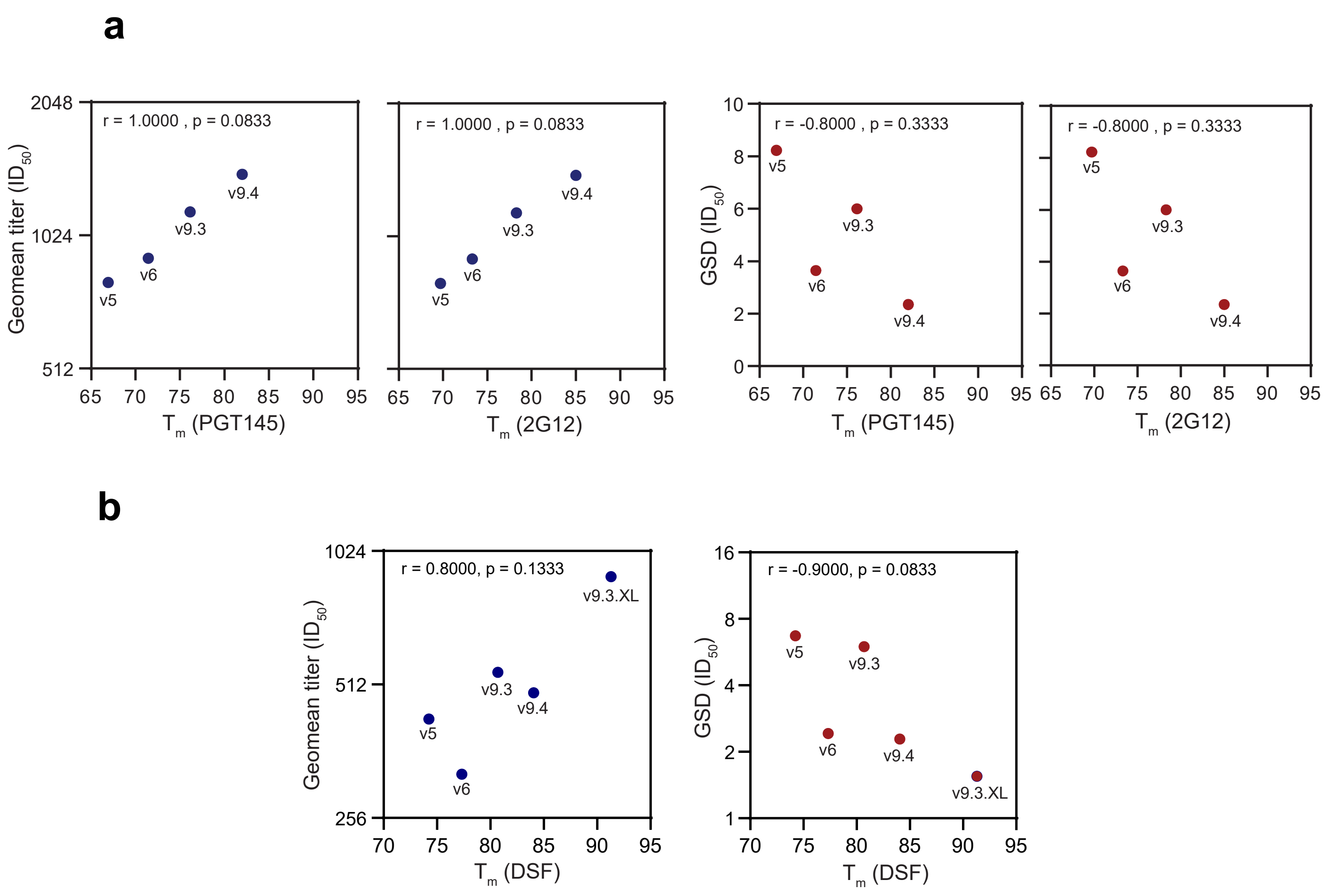

Supplementary Figure 16. Effect of the ultrastabilization on the potency and consistency of autologous NAb responses. **a** Correlation plots between geometric mean (Geomean) ID50 and geometric standard deviation (GSD) of the ID50 against the BG505.dCT virus and the melting temperatures (Tm) of the immunogens, determined by 2G12 and PGT145 thermostability ELISA. **b** Correlation plots between the Geomean and GSD of the ID50 against the full length BG505 pseudovirus and the Tm of the immunogens, determined by nanoDSF. Spearman r and p-values are presented.

Group	Immunogen	Rabbit ID	BG505 WT	BG505 241N/289N	BG505 133aN/136aA	BG505 465N
			Tier 2	Tier 2	Tier 2	Tier 2
			Clade A	Clade A	Clade A	Clade A
			AMC	AMC*	AMC	AMC*
1	SOSIP.v5	UA129	1531	1000	2096	737
		UA130	<20	<20	<20	22
		UA131	258	22	118	61
		UA132	2023	55	1103	603
		UA133	5688	76	3991	2874
2	SOSIP.v6	UA134	<20	32	68	43
		UA135	831	<20	1404	723
		UA136	112	<20	81	220
		UA137	419	<20	927	560
		UA138	795	502	877	681
3	SOSIP.v9.3	UA139	30	75	447	268
		UA140	1334	1498	3373	749
		UA141	92	38	145	131
		UA142	14704	4023	4860	4860
		UA143	127	22	144	95
4	SOSIP.v9.4	UA144	196	84	559	152
		UA145	763	152	892	669
		UA146	435	<20	800	545
		UA147	199	61	138	120
		UA148	1024	255	660	1621
5	SOSIP.v9.3.XL	UA149	1344	37	1342	596
		UA150	1191	262	1271	399
		UA151	283	31	1007	735
		UA152	2157	42	2336	2077
		UA153	1515	525	1944	711
6	SOSIP.v9.3.GM	UA154	48	104	85	48
		UA155	7555	7091	9938	2106
		UA156	<20	<20	<20	<20
		UA157	237	812	1235	574
		UA158	1063	1482	116	380
7	SOSIP.v9.4.GM	UA159	1307	1022	773	107
		UA160	704	732	829	385
		UA161	695	797	941	164
		UA162	704	471	826	250
		UA163	2247	2222	2491	297

Neutralization titers (ID50)
20-40
41-100
101-1000
>1001

Supplementary Figure 17. Midpoint neutralization titers (ID50) for week 22 sera of the immunized rabbits against the autologous BG505/T332N pseudovirus and a panel of mutant viruses. The mutant viruses are pseudotyped with Env containing 241N/289N, 133aN/136aA and 465N mutations. ID50 values were determined in a TZM-bl neutralization assay as described in the methods section. Columns with an asterisk present the average of the ID50 values obtained in two independent replicate assays.

			MLV	BG505/T332N	BG505	N88A	N611A	N625A	N160K	N276Q	N279Q	N280D	G458Y
			Neg. control	Tier 2	Tier 2	Tier 2	Tier 2	Tier 2	Tier 1B	Tier 2	Tier 3	Tier 2	Tier 1A
			-	Clade A	Parental	120-41	120-41	120-41	V2 glycan	CD4bs	CD4bs	CD4bs	CD4bs
Group	Immunogen	Rabbit ID	DUMC	DUMC	DUMC	DUMC	DUMC	DUMC	DUMC	DUMC	DUMC	DUMC	DUMC
3	SOSIP.v9.3	UA139	98	188	195	252	237	229	151	172	138	120	178
		UA142	<20	7474	8074	5459	11304	8978	7776	11098	9744	8490	279
6	SOSIP.v9.3.GM	UA155	36	6520	4279	5937	7607	6251	6494	8780	7761	7878	5478
		UA157	<20	261	319	495	963	506	513	360	373	585	195
		VRC01			0,100	0,104	0,085	0,088	0,117	0,018	0,167	>5	>5
		PG9			0,025	0,028	0,021	0,027	>5	0,025	0,026	0,026	0,022
		PGT128			0,246	0,411	0,081	0,47	0,764	0,455	0,097	0,066	0,019
		PGT151			0,005	0,004	0,036	0,004	0,004	0,004	<0.0023	<0.0023	<0.0023
		VRC34.01			0,073	>5	0,004	0,059	0,059	0,077	0,053	0,041	0,022

Neutralization titers (ID50)	
	20-40
	41-100
	101-1000
	>1001

IC50 against parental BG505 virus

>3-fold reduc on in IC50 compared to parental BG505 virus

Expected result of muta on

Supplementary Figure 18. Midpoint neutralization titers (ID50) for week 22 sera of the UA139, UA142, UA155 and UA156 rabbits against the autologous BG505/T332N pseudovirus, a parental BG505 pseudovirus and a panel of parental-derived mutant viruses containing the indicated mutations (N88A, N611A, N625A, N160K, N276Q, N279Q, N280D, G485Y) that cover several bNAb epitopes on the Env protein. Control bNAbs (VRC01, PG9, PGT128, PGT151 and VRC34.01) are used to map the epitope targeted in the case of a reduction in the titers against a mutant virus.
